# GO-PCA: An Unsupervised Method to Explore Gene Expression Data Using Prior Knowledge

**DOI:** 10.1371/journal.pone.0143196

**Published:** 2015-11-17

**Authors:** Florian Wagner

**Affiliations:** 1 Graduate Program in Computational Biology & Bioinformatics, Duke University, Durham, NC, United States of America; 2 Center for Genomic and Computational Biology, Duke University, Durham, NC, United States of America; Harbin Medical University, CHINA

## Abstract

**Method:**

Genome-wide expression profiling is a widely used approach for characterizing heterogeneous populations of cells, tissues, biopsies, or other biological specimen. The exploratory analysis of such data typically relies on generic unsupervised methods, e.g. principal component analysis (PCA) or hierarchical clustering. However, generic methods fail to exploit prior knowledge about the molecular functions of genes. Here, I introduce GO-PCA, an unsupervised method that combines PCA with nonparametric GO enrichment analysis, in order to systematically search for sets of genes that are both strongly correlated and closely functionally related. These gene sets are then used to automatically generate expression signatures with functional labels, which collectively aim to provide a readily interpretable representation of biologically relevant similarities and differences. The robustness of the results obtained can be assessed by bootstrapping.

**Results:**

I first applied GO-PCA to datasets containing diverse hematopoietic cell types from human and mouse, respectively. In both cases, GO-PCA generated a small number of signatures that represented the majority of lineages present, and whose labels reflected their respective biological characteristics. I then applied GO-PCA to human glioblastoma (GBM) data, and recovered signatures associated with four out of five previously defined GBM subtypes. My results demonstrate that GO-PCA is a powerful and versatile exploratory method that reduces an expression matrix containing thousands of genes to a much smaller set of interpretable signatures. In this way, GO-PCA aims to facilitate hypothesis generation, design of further analyses, and functional comparisons across datasets.

## Introduction

Genome-wide expression profiling, or *transcriptomics*, is a highly popular approach for obtaining a systematic view of the molecular differences and similarities among cells, tissues, tumor biopsies or other biological specimen. The success of transcriptomics is based on advances in microarray and high-throuhgput sequencing technologies, which have led to reductions in costs and improved measurement accuracies. Currently, the development of single-cell methods is promising a dramatic increase in the spatial resolution of transcriptomic data [1] (see e.g. [2–4], recent applications of single-cell transcriptomics in the fields of developmental biology, cancer research, and stem cell biology, respectively).

Considering the rapid pace and low cost at which large-scale transcriptomic datasets can be produced, data analysis often presents a significant bottleneck. The machine learning literature offers a plethora of methods for unsupervised learning, which have been adopted to various degrees for the exploratory analysis of gene expression data. Popular approaches include principal component analysis (PCA) [5], hierarchical clustering [6], k-means clustering, consensus clustering [7], non-negative matrix factorization (reviewed in [8]), mixture models (e.g., [9]), and many others. These methods can be characterized as *generic*, in that they operate based on general principles (e.g., prinicipal components are uncorrelated and capture maximum amounts of variance), and do not take any specific biological aspects of the data into account.

While applications of the aforementioned methods have led to profound insights into biological processes (e.g., the identification of clinically relevant cancer subtypes [10, 11]), arriving at such results typically requires significant human effort combined with expert knowledge, and can be fraught with difficulties. In many cases, the data contain significant but unknown biases which can obscure interesting signals and create spurious results (e.g., batch effects [12]). Furthermore, the output of unsupervised methods often consists of clusters or factors containing hundreds of genes, which are difficult to interpret and necessitate further analysis before any biological intuition can be applied.

These challenges motivate the development of more specialized tools for the exploratory analysis of transcriptomic data that 1) improve the detection of biologically relevant patterns, 2) confer robustness with respect to technical artifacts, and 3) yield readily interpretable results that facilitate hypothesis generation. The incorporation of *prior knowledge* into unsupervised algorithms provides a major opportunity for achieving these goals. In principle, prior knowledge can bias the analysis in favor of biologically plausible results, thereby reducing the influence of extraneous biases such as batch effects, which do not exhibit biologically meaningful patterns. It can also help provide meaningful labels for discovered patterns, which in turn facilitates the interpretation of results [13].

In light of the intuitive appeal of this idea, as well as its highly successful application in supervised settings [14], there exist surprisingly few methods that exploit prior biological knowledge in a general unsupervised setting. Several methods have been designed for the narrow task of identifying regulatory relationships ([15] and ref. 11–14 in [13]). For more general purposes, it has been proposed to adjust the distance metric used in hierarchical clustering by a term that quantifies similarity of GO or KEGG annotations between pairs of genes, with a tuning parameter allowing for a flexible trade-off between knowledge-based and data-driven analysis [16, 17]. Annotation-based adjustments have also been proposed for use in k-means/k-medioid clustering [18–20] and mixture models [21].

The method proposed here relies on PCA, one of the most versatile unsupervised methods, and uses prior knowledge in the form of gene ontology (GO) annotations from the UniProt-GOA database [22]. However, rather than using these annotations to adjust an internal metric, the method adopts a two-step approach. PCA is performed first, and then each principal component is tested for whether it is driven by functionally related genes. This leads to the definition of *signatures*, consisting of small sets of genes that are both strongly correlated in the input data, as well as functionally related based on their GO annotations. These signatures are visualized in a *signature matrix*, which can then serve a starting point for further data exploration.

## Results

### GO-PCA combines principal component analysis (PCA) with nonparametric GO enrichment analysis

**Fig 1:**
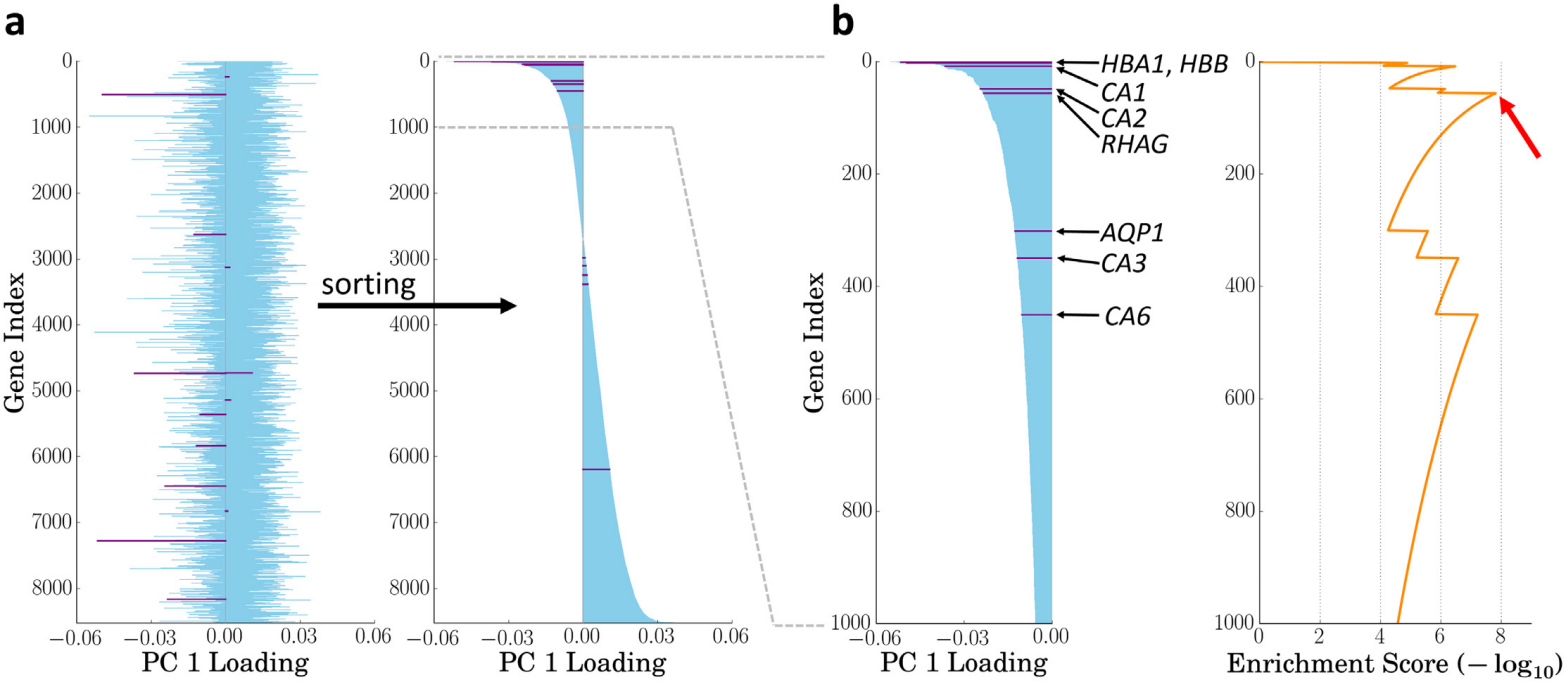
GO-PCA schematic. The figure provides a simplified illustration of the key idea behind the method using PC 1 of the DMAP dataset and the GO term “bicarbonate transport” (BT; GO:0015701) as an example. All genes annotated with the GO term are highlighted in purple. **a** After performing PCA on the gene expression matrix, genes are ranked according to their PC loadings. **b** The ranked list of genes is tested for GO enrichment using the XL-mHG test. In this example, the highest enrichment score (red arrow) is associated with the first five BT genes (*HBA1*, *HBB*, *CA1*, *CA2*, and *RHAG*; *p* = 8.3 × 10^−8^). These five genes are then used to generate a signature labeled with “bicarbonate transport” (red star in Fig 2). See Methods for details.

In order to facilitate exploration of transcriptomic data using prior knowledge, I sought to design a method that would systematically search all major axes of variation for small groups of genes that are both strongly correlated and functionally related, and then present the results in an easily interpretable fashion. To this end, I developed the GO-PCA algorithm, named after its two building blocks, PCA [5], and GO enrichment analysis [23]. GO-PCA first performs PCA on the expression matrix and determines the number of relevant principal components (PCs) using a permutation test. It then tests each PC for enrichment of functionally related genes. More formally, for each PC, genes are first ranked by their loadings (see Fig 1a). Given the ranked list of genes obtained from a particular PC, GO-PCA then uses the XL-mHG test [24] to detect GO terms (i.e., sets of functionally related genes) which are significantly enriched at the top of that list (see Fig 1b). The XL-mHG is a simple extension of the *minimum hypergeometric* (mHG) test [23, 25], which is a powerful nonparametric test for enrichment in ranked binary lists that produces an exact p-value. Since GO-PCA tests thousands of GO terms in this way, it applies a stringent Bonferroni correction to the p-values obtained. For each significantly enriched term, the genes underlying the enrichment are used to derive an expression signature based on standardized expression values. The primary output of GO-PCA is a *signature matrix* that provides a readily interpretable view of biological heterogeneity in the data. GO-PCA also prioritizes and filters the GO terms it finds to be enriched, in order to limit signature redundancy. The reader may refer to the Methods section for a detailed description of the full algorithm.

### Application of GO-PCA to a diverse panel of hematopoietic cell types recovers known lineage characteristics

As a first test of my method, I aimed to apply GO-PCA to a highly heterogeneous dataset composed of biologically well-defined subsets of samples. For such a dataset, GO-PCA should ideally generate a compact set of signatures, each associated with a specific subset, and with a label reflecting a biological characteristic specific to this subset. I therefore applied GO-PCA to a dataset comprising 211 samples, representing 38 distinct cell populations from 15 hematopoietic lineages [26] (this dataset will henceforth be referred to as DMAP). GO-PCA tested the first 15 PCs, and produced a signature matrix with 50 signatures containing between 5 and 43 genes (see S2 Fig). As expected based on the composition of the dataset, many signatures were derived from GO terms representing immune-related functions. Furthermore, the genes within most signatures were strongly correlated with each other, as evidenced by median pairwise correlation coefficients of 0.5 or greater (see S3 Fig). Each signature was expressed in only a subset of samples, often with standardized expression levels of 2 or greater. At the same time, virtually all samples exhibited high expression of at least one of the signatures.

**Fig 2:**
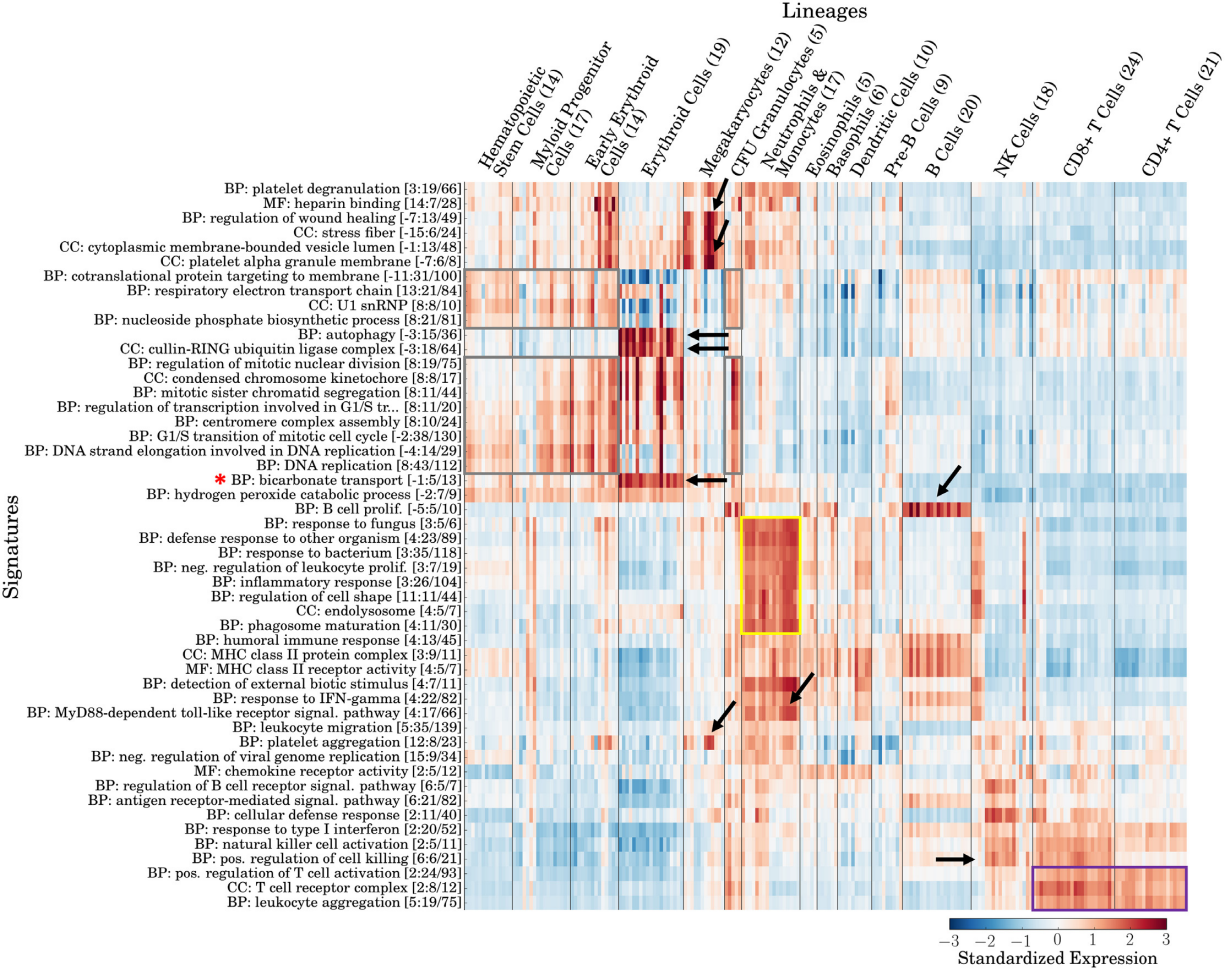
Validation of GO-PCA using a dataset of 211 human transcriptomes representing diverse hematopoietic cell types (DMAP; [26]). Shown is a heat map of the signature matrix generated by GO-PCA, with signatures (rows) ordered using hierarchical clustering with correlation distance and average linkage, and samples (columns) ordered according to their known lineage identities. Arrows and boxes indicate specific associations between signatures and lineages discussed in the text. Signature labels (left) indicate the name of the GO term the signature is derived from. “BP”, “MF”, and “CC” refer to “biological process”, “molecular function”, and “cellular component”, the three main branches of the gene ontology. The three numbers in parentheses indicate, respectively, 1) the principal component that the GO term was found to be enriched in (with a negative sign indicating enrichment among the genes with the *lowest* loadings), 2) the number of genes in the signature, and 3) the total number of genes in the analysis that were annotated with the GO term. Signature expression levels are calculated as the unweighted average over the standardized expression levels of each gene in the signature (see Methods for details).

In order to examine whether labels and expression patterns of the signatures generated by GO-PCA agreed with hematopoietic lineages and their known biological characteristics, I grouped the samples in the signature matrix by their lineage identities (see Fig 2). This immediately revealed several strikingly specific associations: For example, two signatures, derived from the GO terms “bicarbonate transport” (BT) and “autophagy”, respectively, were strongly and exclusively associated with the erythroid lineage (red blood cells). Both of these functional categories match unique biological characteristics of erythrocytes, namely their ability to transport carbon dioxide [27], and the degradation of their mitochondria through a type of autophagy termed “mitophagy” [28]. This autophagy signature was also almost perfectly correlated with an 18-gene “cullin-RING ubiquitin ligase complex” (ULC) signature (see S4a and S4b Fig), pointing towards a role of ubiquitin ligases in reticulocyte development (see S3 Text). It is worth noting that the genes in the BT and autophagy signatures were associated with only 0.5% and 0.2% of the total variance in the data, respectively, highlighting GO-PCA’s ability to identify small, specific, and functionally relevant signatures against a highly heterogeneous background.

Specific associations of signatures with other cell types were also readily spotted: Most samples of megakaryocytes, which serve to produce platelets [29], were associated with “platelet alpha granule membrane”, “platelet aggregation” and “regulation of wound healing” signatures. Likewise, B cells were strongly associated with a “B cell proliferation” signature, whereas the neutrophil / monocyte lineage was associated with multiple signatures (see yellow box in Fig 2) which possibly reflected their related immunological roles in the innate immune system [30, 31]. For example, the signatures labeled “endolysosome” and “phagosome maturation” matched their phagocytotic capabilities, and the signatures “response to fungus” and “response to bacterium” (the latter encompassing 35 genes) agreed with their importance in defending the body against fungal and bacterial infections. A 17-gene “MyD88-dependent toll-like receptor signal. pathway” (see S4c Fig) was also specifically expressed in this lineage, likely reflecting the fact that toll-like receptors (TLRs) are important pattern recognition receptors for phagocytes [31]. For example, the genes *TLR2* and *TLR4* were both part of this signature, and are known to play important roles in monocytes ([32] p. 43) and neutrophils [33]. These functional matches between signature labels and their associated lineages demonstrated that many of the signatures generated by GO-PCA were both interpretable and specific.

On top of these highly specific associations, some signatures showed broader associations: For example, two “positive regulation of T cell activation” and “T cell receptor complex” signatures were associated with all samples from the T cell lineage, regardless of whether they were CD4+ or CD8+ (see purple box in Fig 2). Furthermore, NK cells and CD8+ T cells, but not CD4+ T cells, shared a “positive regulation of cell killing” signature, in agreement with their cytotoxic capabilities. Another group of signatures, related to cell cycle and metabolic processes, was associated with the lineages representing stem cells or partially differentiated cell types (i.e., hematopoietic stem cells, myeloid progenitor cells, early erythroid cells and CFU-granulocytes; see gray boxes in Fig 2). Since these populations can be expected to exhibit elevated proliferation rates, these associations could be interpreted to reflect an increased mitotic activity of the respective cells. Curiously, an eight-gene “U1 snRNP” (U1 small nucleolar ribonucleoprotein) signature exhibited a similar stem cell-specific expression pattern. Variant U1 small nucleolar RNAs (snoRNAs) have been shown to be specifically expressed in human embryonic stem cells [34], and the observation of a stem cell-specific pattern of the corresponding ribonucleoproteins here similarly suggests a role for U1 SNPs in stem cell maintenance. These examples of signatures with broader associations demonstrate that the signatures generated by GO-PCA enabled examination of the data at different levels of granularity.

In summary, the expression patterns of the signatures generated by GO-PCA without knowledge of lineage identities were largely consistent with the observation of five “main” lineages by Novershtern et al., comprising hematopoietic stem and progenitor cells (HSPCs), erythrocytes (ERY), granulocytes/monocytes, B cells, and T cells. The signature matrix greatly facilitated functional annotation of expression patterns, and revealed complex relationships among subsets of samples that are sometimes difficult to appreciate based on the output of generic unsupervised methods (see e.g., Figure S1A in [26]).

### Bootstrap analysis reveals robustness of GO-PCA signatures

**Fig 3:**
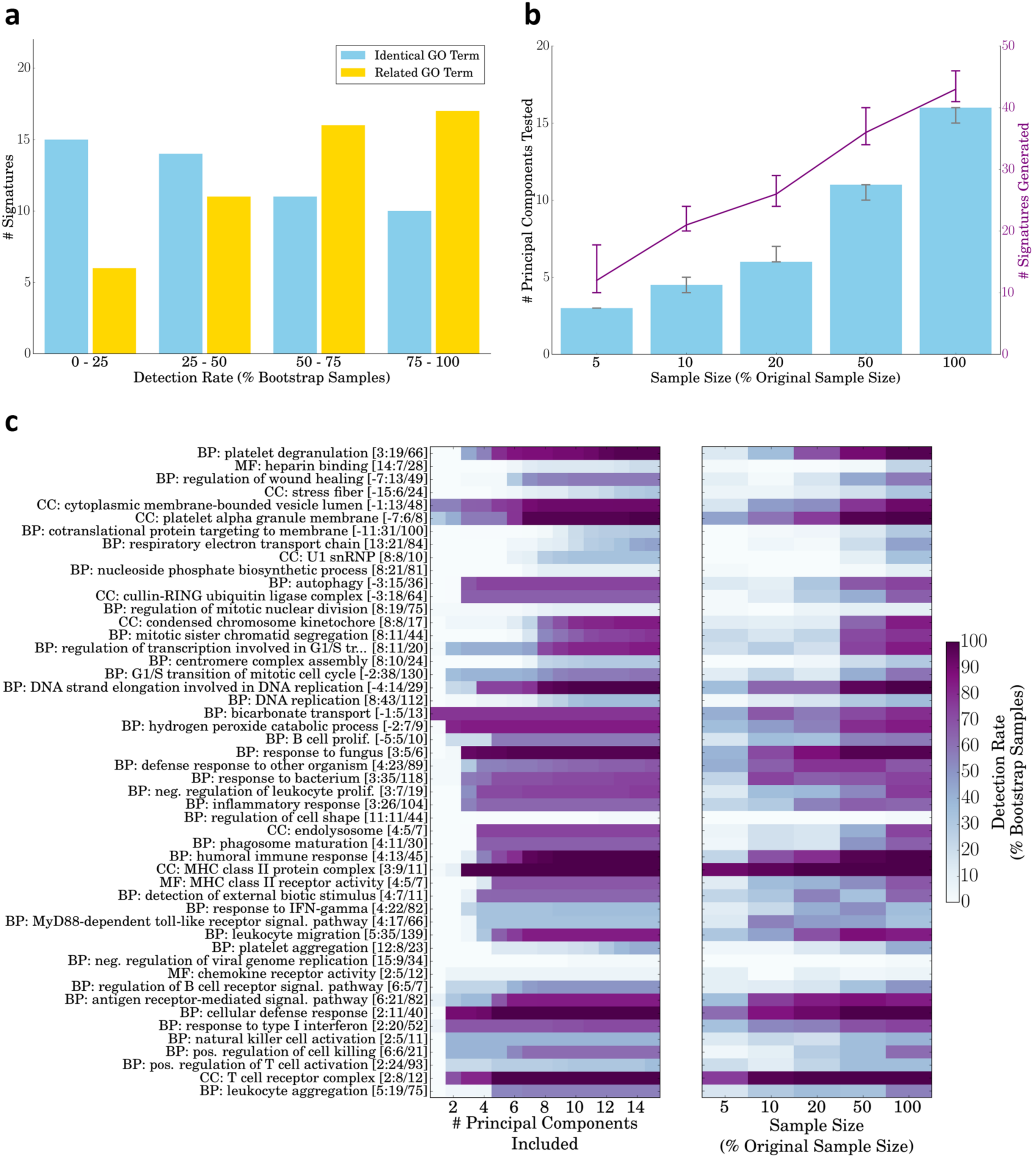
Analysis of the robustness of DMAP GO-PCA signatures using bootstrapping. **a** Detection rates of the 50 DMAP signatures generated by GO-PCA (see Fig 2). GO-PCA was applied to 50 bootstrap samples with the same size as the original dataset (*n* = 211). In each analysis, a signature from the original analysis was counted as detected if there existed a signature based on the exact same GO term (yellow bars), or based on either the same GO term or any “related” GO term (blue bars). Related GO terms were defined as all ancestor and descendent terms in the Gene Ontology. This figure panel was generated using the GO-PCA script gopca_plot_bootstrap_signature_recovery.py. **b** Number of principal components tested and signatures generated for bootstrap samples with different sample sizes. For each size, GO-PCA was applied to 50 bootstrap samples of that size. Shown are the median values for each measure, and error bars indicate the inter-quartile range. This figure panel was generated using the GO-PCA script gopca_plot_bootstrap_numbers.py. **c** Bootstrap detection rates of all DMAP GO-PCA signatures, as a function of the number of PCs included in the analysis (left), and as a function of the size of the bootstrap samples (right). This figure panel was generated using the GO-PCA scripts gopca_plot_bootstrap_pc_matrix.py and gopca_plot_bootstrap_sample_size_matrix.py.

Since GO-PCA applies a series of tests and filters in order to generate signatures, I decided to test the robustness of GO-PCA signatures and their dependency on sample size using bootstrapping (sampling with replacement, [35]). I first sampled 50 datasets with the same size as the original dataset (*n* = 211). 21 of the 50 signatures generated in the original analysis had a bootstrap detection rate of at least 50%, meaning that in at least 50% of the simulated datasets, GO-PCA generated signatures based on the exact same GO term. I then relaxed this requirement, so that a signature was counted as detected if a signature that was based on a “related” GO term was present, defined as any ancestral or descendant term in the Gene Ontology. (Note that this definition was not as broad as it might appear, as only GO terms with 200 or fewer genes were included in the analysis to begin with; see Methods.) Using this relaxed criterion, the number of signatures with a detection rate of at least 50% rose to 33, and most of the remaining signatures were detected in at least 25% of datasets (see Fig 3a). I next used bootstrapping to sample datasets with sizes corresponding to 5–50% of the original dataset. Application of GO-PCA to these smaller datasets resulted in the selection of fewer PCs for testing, and led to the generation of fewer signatures (see Fig 3b). This behavior made intuitive sense, as I expected smaller datasets to exhibit fewer meaningful principal components. As an example, for *n* = 10, GO-PCA tested only the first three PCs in the majority of cases, and generated a median number of 12 signatures. I also noted that for each sample size tested, the number of signatures generated for the bootstrap samples was quite similar. These results showed that both the number and the functional categories of signatures generated by GO-PCA were relatively robust, and that GO-PCA was able to automatically adjust the number of PCs to test based on the sample size.

I next aimed to examine the results of performing GO-PCA on bootstrap samples in more detail. To test how much the generation of individual signatures depended on specific PCs, I used the bootstrap samples with full sample size (*n* = 211) to calculate how signature detection rates changed when only signatures generated using the first *n* principal components were included in the analysis. Many signatures showed a clear association with specific PCs, indicated by a sharp increase in the detection rate as soon as the signatures from a particular PC were included. For example, the “autophagy” signature appeared robustly associated with PC 3 Fig 3c, left heat map). The results also showed that some signatures, such as “condensed chromosome kinetochore” were exclusively generated based on higher PCs. I next used the bootstrap samples with smaller sample sizes to examine how the detection rate of individual signatures depended on the sample size. This revealed that while the “T cell receptor complex” (TCR) and “MHC class II protein complex” (MHC) had a very high detection rate even in datasets with only 10 samples, other signatures, including the “autophagy” signature discussed earlier, required a sample size of at least 50% of the original for robust detection. These differences are obviously related to the fact that the TCR and MHC signatures showed broad expression (see Fig 2), so that even bootstrap samples with *n* = 10 or *n* = 20 will include a few samples expressing both high and low levels of those genes. Interestingly however, there were also cases in which signatures with similar expression patterns had very different sample size dependencies. For example, while both signatures were most highly expressed in erythrocytes, the “bicarbonate transport” signature could be detected with a sample size of 10%, whereas the “cullin-RING ubiquitin ligase” signature required the original sample size for robust detection. In summary, this analysis provided a quantitative view of the robustness of individual signatures, including their association with specific PCs, as well as their dependencies on sample size.

### Application of GO-PCA to a large panel of mouse immune cell types recovers known lineage characteristics

To test the performance of GO-PCA on non-human expression data, I next applied the method to a panel of 650 transcriptomes representing 214 cell populations from mouse [36]. I obtained one transcriptome for each population by processing the raw microarray data and averaging expression levels across replicates (see Methods for details). I further grouped cell populations into 15 lineages or sub-lineages (e.g., neutrophils, dendritic cells, and CD4^+^T cells), according to the sample annotations [36]. (This dataset is henceforth referred to as IGP1.) In comparison to DMAP, IGP1 comprises more than five times as many cell populations, each represented by a robust average expression profile obtained from between three to seven replicates. Furthermore, IGP1 includes an outgroup of ten non-hematological stromal cell types.

The application of GO-PCA to IGP1 resulting in the testing of the first 21 PCs, and led to the generation of 89 signatures, which contained between 5 and 66 genes (see S5 Fig). The genes within most signatures again had high internal correlations (see S6a Fig; median value = 0.55), and bootstrap analysis showed their robustness with respect to GO terms and their overall number (see S6b and S6c Fig). The significantly larger number of signatures, as compared to the DMAP analysis, likely reflected the greater diversity and resolution of the dataset (see above). Some signatures were highly correlated, but overall the signatures had diverse expression patterns covering all samples.

**Fig 4:**
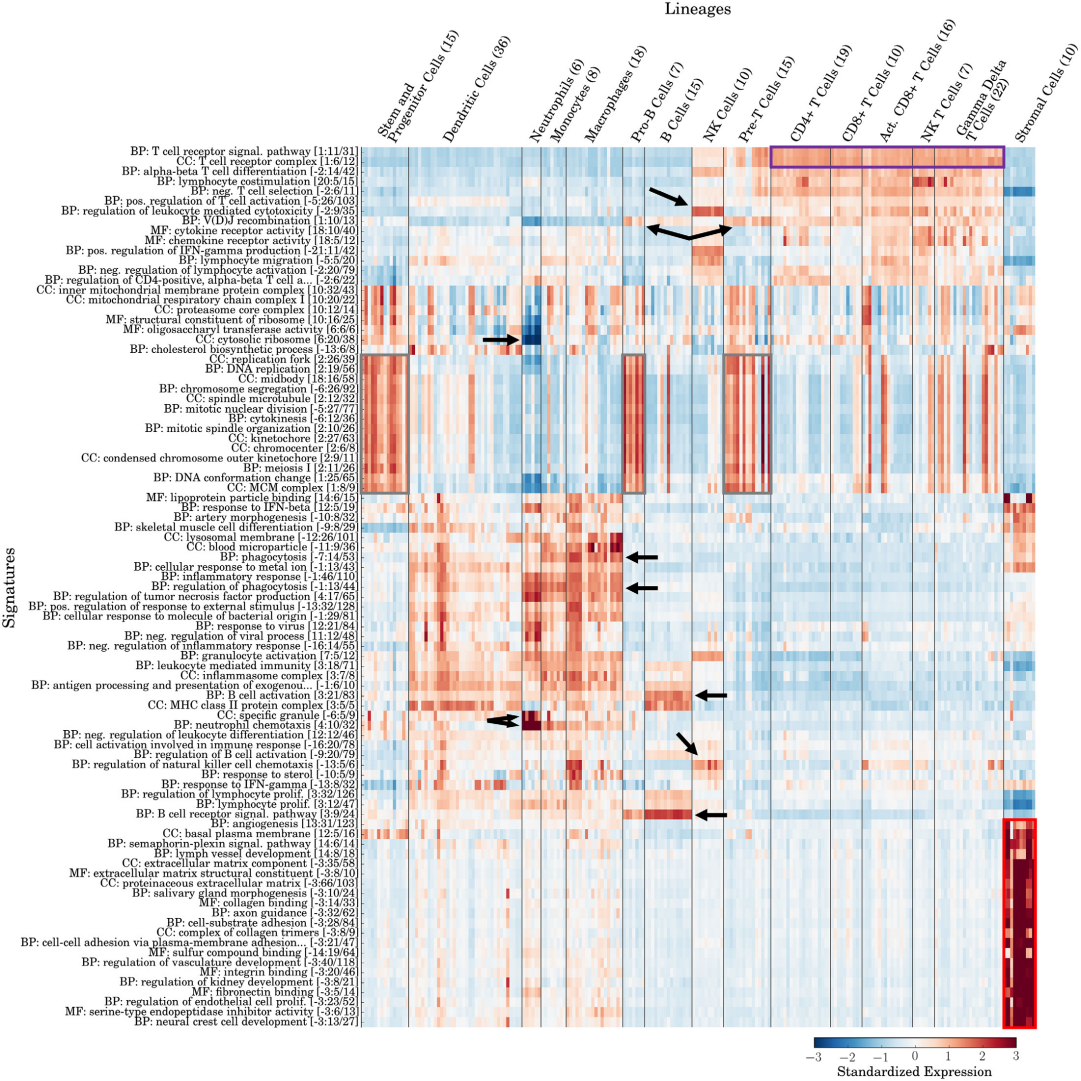
Application of GO-PCA to 214 mouse transcriptomes representing diverse immune and stromal cell types (IGP1). [36] Signatures are labeled and ordered as in Fig 2. Arrows and boxes indicate associations discussed in the main text. Samples are ordered according to their known lineage identities.

To assess whether signatures agreed with known lineage characteristics, I again re-grouped samples according to their lineage identities (see Fig 4). As expected, the stromal cells formed an outgroup that was associated with multiple highly correlated signatures, mostly related to extracellular matrix (ECM) components, blood/lymph vessel development, and neural development (see red box in Fig 4). In addition to identifying and characterizing these “outgroup” samples, GO-PCA again produced many signatures that precisely matched individual immune cell lineages: For example, two “B cell receptor signaling pathway” and “B cell activation” signatures were specifically expressed in B cells. Similarly, two signatures labeled “regulation of leukocyte mediated cytotoxicity” and “regulation of natural killer cell chemotaxis” were associated with NK cells. As in the analysis of DMAP, all types of T cells were associated with T cell receptor-related signatures (see purple box in Fig 4), and several signatures related to cell division (see gray boxes in Fig 4 and S7d Fig) were strongly associated with the three lineages corresponding to developmental precursor stages: 1) stem and progenitor cells, 2) pro-B cells, 3) pre-T cells. Neutrophils and monocytes/macrophages again shared expression of several signatures such as “regulation of phagocytosis”, matching their shared status as phagocytes. However, another “phagocytosis” signature had markedly lower expression in neutrophils. Two signatures were uniquely associated with neutrophils: “neutrophil chemotaxis” (see S7a Fig) and “specific granule”. Specific granules are a prominent feature of neutrophils, ([37] p. 128). At the same time, neutrophils exhibited extremely low expression of a “cytosolic ribosome” signature (see S7b Fig), which agreed with the observation that mature neutrophils have few ribosomes ([38] p. 66). In summary, GO-PCA generated specific and appropriately labeled signatures for multiple hematopoietic lineages in mouse, and it was able to do so even in the presence of an outgroup of samples with no relationship to the rest of the data. A case where a signature expression profile defied lineage boundaries was that of “V(D)J recombination” (see S7c Fig), which appeared most strongly expressed in Pro-B cells and Pre-T cells, corresponding to the cell types in which V(D)J recombination and assembly of B and T cell receptors is known to occur [39]. The ability of GO-PCA to generate a signature which recovered a specific biological process shared between a small set of samples from two different lineages provided additional evidence for its effectiveness in exploring heterogeneous expression data.

In comparing the GO-PCA results from the human (DMAP) and mouse (IGP1) datasets, the similarities between some signatures in terms of their labels and behaviors across cell types were striking. For example, for the two datasets, GO-PCA identified 12 and 14 signatures, respectively, associated with stem cells or partially differentiated cells (gray boxes in Figs 2 and 4). Among those, signatures with labels pertaining to chromosome/chromatid segregation, kinetochore, cell division and DNA replication were present in both human and mouse. Both analyses furthermore identified a “T cell receptor complex” signature associated with T cells, as well as similar B cell-specific signatures (“B cell proliferation” vs. “B cell activation”). Likewise, signatures related to phagocytosis with specific expression in the monocyte and neutrophil lineages were identified in both analyses. In all these cases, GO-PCA therefore found identical or highly similar GO terms to be enriched in both datasets. Even if the lineage identities had not been known in advance, these signatures would have suggested a functional correspondence between the associated samples from the two species.

### Application of GO-PCA to glioblastoma data results in subtype-specific signatures

**Fig 5:**
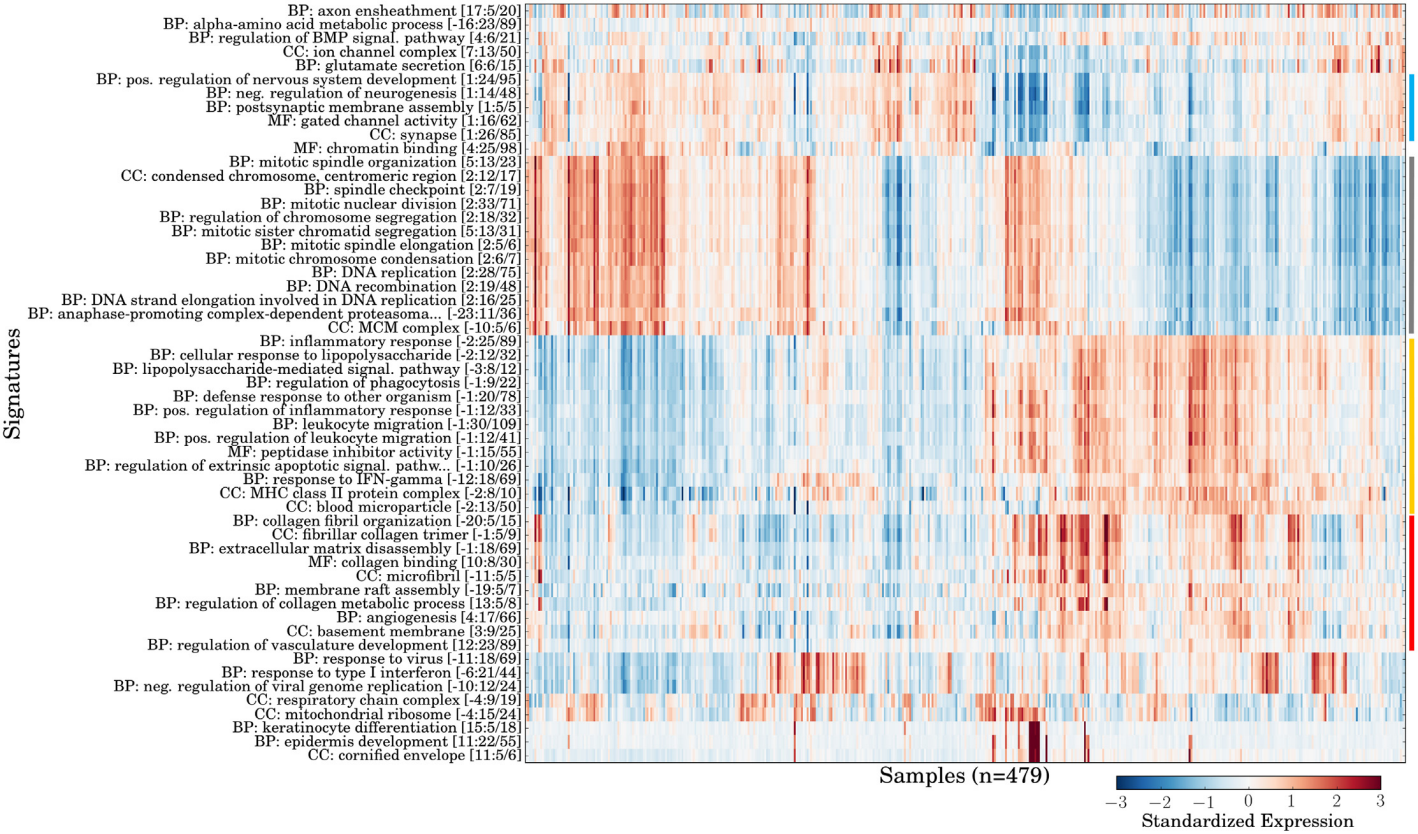
Application of GO-PCA to 479 primary glioblastomas (GBM). Shown is the signature matrix generated by GO-PCA as a heat map. Signatures are labeled and ordered as in S2 Fig, and samples are ordered using hierarchical clustering with correlation distance and average linkage. Colored bars at the side indicate the four main groups of signatures discussed in the text (blue = neuronal; gray = proliferative; yellow = immunological; red = extracellular matrix (ECM)-related). This figure (without colored bars) was generated using the GO-PCA script gopca_plot_signature_matrix.py.

After validating the ability of GO-PCA to generate meaningful signatures for different hematopoietic lineages in both human and mouse, I aimed to test whether GO-PCA could also facilitate exploration of human tumor expression data. To this end, I applied GO-PCA to 479 transcriptomes of glioblastomas from patients diagnosed with primary glioblastoma (GBM; this dataset is henceforth referred to as GBM). GBMs are highly aggressive brain tumors, and patients have a median survival time of under 14 months [40]. Application of GO-PCA to the GBM dataset resulted in the testing of 30 PCs and the generation of 55 signatures. Most signatures again showed strong internal correlations of 0.5 or greater (see S8 Fig; median value = 0.52), and were robust by bootstrap analysis, with 35 signatures exhibiting a detection rate of above 50% (when counting related GO terms, see S9a Fig). The large number of PCs tested seemed to be largely a result of the large sample size (*n* = 479), as bootstrapping with 50% of the sample size (*n* = 239) resulted in the testing of an average of only 25 components (see S9b Fig), which was comparable to the results obtained for the IGP1dataset (see above). The signature matrix (see Fig 5) indicated the presence of four groups of functionally related and correlated signatures. The functional categories represented by those groups of signatures could be broadly described as neuronal, proliferative, immunological, and extracellular matrix (ECM)-associated, respectively. Interestingly, all four groups contained signatures that were generated based on either the first or the second PC, suggesting that their expression patterns were each related to one of the major axes of variation in the data. To better understand how these signatures were related to each other, I examined their pair-wise correlations (see S10 Fig). This revealed strong correlations between signatures in the immunological and ECM-associated groups. In contrast, these two groups were both strongly anti-correlated with the neuronal group. The proliferative group was not strongly correlated with the other groups, except for its anti-correlation with the immunological group. In summary, the four main groups of signatures were neither perfectly correlated with each other, nor completely mutually exclusive, and the behavior of the other signatures (e.g., “response to type I interferon”) added additional complexity to this picture.

Surprisingly, three of the four signature groups resembled groups obtained in the previous analyses of the DMAP and IGP1 datasets, based on a comparison of the GO term names: First, terms in the proliferation group (e.g., “DNA replication”), were partly identical to the ones found for stem cells and partially differentiated cells in those analyses. Secondly, terms in the immunological group (e.g., “inflammatory response”, “regulation of phagocytosis”, and “MHC Class II protein complex”) were identical to some of those previously generated for neutrophils, macrophages, and antigen-presenting cells. Lastly, the GO terms from the ECM group were either identical (“collagen binding”, “angiogenesis”) or very similar (e.g., involving references to the extracellular matrix). Therefore, signatures in the immune group could represent different types of immune cells infiltrating the tumor in the affected samples, and the (strongly correlated) expression of the ECM signatures could represent the presence of stromal tissue. However, more careful analyses of individual signature genes, as well as validation with external methods such as immunohistochemistry staining of tissue sections would be required to conclusively connect signature expression with admixture of non-malignant cells. Based on the anti-correlation of proliferative and immunological signatures, it was also unclear if variable proliferation signature expression was associated with differences in the mitotic activities of tumors, or whether it was an artifact resulting from the variable presence of infiltrating immune cells. Since it seems reasonable to assume that these cells exhibit significantly lower proliferation rates than the tumor cells, a larger proportion of immune cells could “dilute” the tumor-specific expression of genes in these signatures.

**Fig 6:**
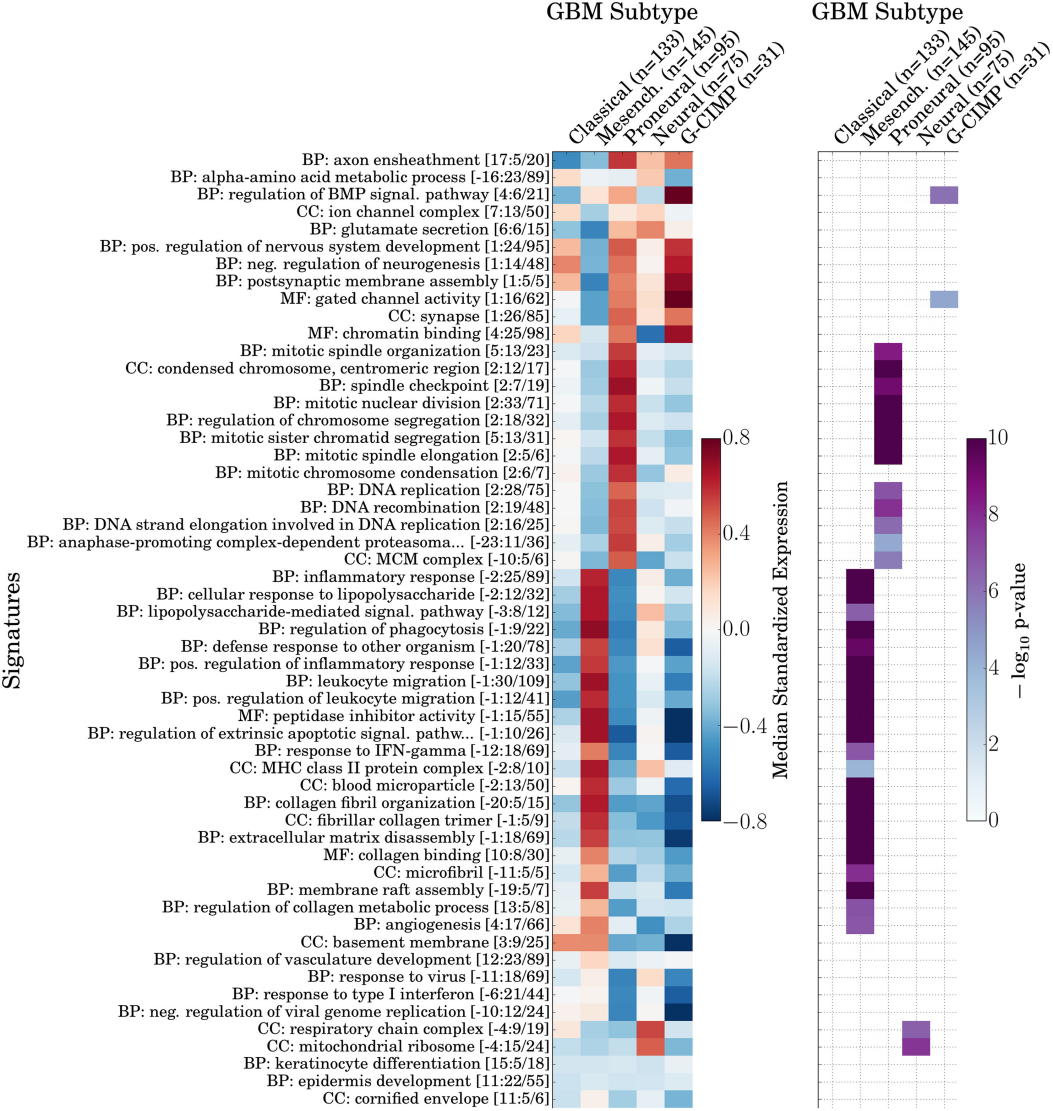
Associations between signatures generated by GO-PCA analysis of the GBM dataset, and five previously defined GBM subtypes [40]. Signatures are shown in the same order as in Fig 5. Left panel: Heat map showing median signature expression levels for each signature in each GBM subtype. Right panel: Heat map showing significance of association, as determined by two-sided Mann-Whitney U tests (see Methods). Only associations with p-values significant at the *α* = 0.05 significance level after Bonferroni correction are shown.

To assess whether the signatures generated by GO-PCA were associated with previously defined GBM subtypes, I used the classifications of the samples into five subtypes, as provided by Brennan et al. Four of these subtypes (Classical, Mesenchymal, Proneural, and Neural) were originally defined based on consensus clustering of gene expression data, and shown to correlate with different chromosomal aberrations and mutations [41]. The fifth subtype, which is rare among patients with primary GBM, was originally defined based on the analysis of DNA methylation patterns, and shown to exhibit a “GBM CpG island methylator phenotype” (G-CIMP) that was associated with significantly better disease outcomes [42]. I calculated median signature expression levels for each subtype, and found four of them to be significantly associated with at least one signature (see Fig 6). Conversely, most signatures were associated with one subtype. Specifically, signatures in the immune system and ECM groups were strongly and significantly associated with the Mesenchymal subtype, and signatures in the proliferation group were similarly strongly associated with the Proneural subtype. In contrast, the neuronal group of signatures was strongly expressed in both the Proneural and the G-CIMP subtype. Furthermore, I found two mitochondrial signatures that were significantly associated with the Neural subtype, as well as two signatures that were most strongly associated with the G-CIMP subtype. In combination, the neuronal and proliferation signatures appeared to capture similarities and differences between the Proneural and G-CIMP subtypes: While both exhibited expression of neuronal genes, they had very different expression profiles for the proliferation signatures. This appeared to agree with the fact that samples with the G-CIMP subtype were originally classified as the Proneural subtype, suggesting a certain degree of relatedness [42]. In summary, these results demonstrated that GO-PCA was able to recover signatures associated with previously described functional GBM subtypes.

## Discussion

### GO-PCA as a new method for the exploratory analysis of gene expression data

The high-dimensional and heterogeneous nature of transcriptomic data often makes it difficult to interpret the output of generic unsupervised algorithms, and technical artifacts can lead to the identification of biologically irrelevant clusters or factors [12] that further complicate the analysis. Methods incorporating prior knowledge address these challenges by focusing on patterns that are more likely to be biologically meaningful, leading to results that are more relevant and interpretable [13]. Here, I introduced an exploratory method that first performs PCA to identify all major axes of variation, and then uses GO enrichment analysis as a way to test for enrichment of functionally related genes driving each PC. While the mHG algorithm has previously been used for GO enrichment analysis [23], GO-PCA is the first method to perform nonparametric GO enrichment analysis on genes ranked by PC loadings (to the best of my knowledge).

GO-PCA relies on the notion that GO terms enriched among genes driving particular PCs represent the biological processes with respect to which samples in the dataset differ from one another. GO-PCA takes this idea one step further, by using significantly enriched GO terms to define signatures, consisting of functionally related and strongly correlated genes. I believe that these signatures exhibit several properties that are desirable in an exploratory setting: First, the association of signatures with individual PCs provides information about whether they represent a major source of variation (if they are associated with one of the first PCs), or a more subtle signal (if they are associated with a “higher” PC). Secondly, the number of samples affected and the relative magnitude of the differences can be judged directly from the signature matrix. Third, while GO-PCA’s filtering rules prioritize the GO terms with the strongest enrichment, they sometimes allow for the inclusion of overlapping terms (see Methods). These then offer “alternative explanations”, which help to reduce the likelihood of drawing wrong conclusions based on the enrichment of a single term that might not accurately reflect the underlying biological process. Fourth, the number of genes that contribute to a given signature is relatively small (typically 5–25, and rarely more than 50). Since the expression value of a signature is furthermore calculated as the unweighted average expression of its genes (after standardization), this provides transparency in terms of how signature expression values are related to the underlying raw data. Fifth, averaging the standardized expression values of several genes imparts the signature with a certain level of robustness with respect to measurement errors and the exact set of genes included. Finally, by assessing the correlation between signatures, researchers can explore how different sources of heterogeneity are related in the data.

### The trade-off between bias and interpretability in knowledge-based methodologies

Prior knowledge can be understood as a bias that is introduced to the unsupervised analysis. Some methods that incorporate prior knowledge enable control over the strength of this bias using an explicit tradeoff parameter [13]. GO-PCA is not designed as a “flexible-tradeoff” method. Its first step, PCA, does not incorporate any prior knowledge, while its second step, GO enrichment analysis, completely ignores signals that do not follow any known functional relationships. As a result, GO-PCA usually excludes the measurements of the majority of genes from its final output. This certainly incurs a risk of overlooking important biological heterogeneity in the data due to incomplete GO annotations, and can obscure problems such as batch effects that researchers might want to known about. It is therefore advisable to not exclusively rely on annotation-dependent methods such as GO-PCA to analyze data. However, the benefits of GO-PCA’s reliance on prior knowledge are equally clear: Compared to most generic methods, GO-PCA’s output is much more easily interpretable. Indeed, the raw GO-PCA output for the GBM dataset not only enabled the definition of functional groups of signatures associated with previously defined GBM subtypes, but also facilitated an intuitive comparison with unrelated human (DMAP) and mouse expression data (IGP1).

### Application of GO-PCA for the exploratory analysis of tumor expression data

The analysis of the GBM dataset presented here can only be considered as a starting point for further applications of GO-PCA to tumor expression data in general, and GBM expression profiles in particular. As for other cancers, the exploratory analysis of GBM expression data has relied heavily on generic clustering algorithms (e.g., [41, 43]). GO-PCA could serve to address three limitations of those approaches: First, GO-PCA represents a systematic way of generating functional annotations for gene expression patterns, making a manual annotation of clusters largely unnecessary. While the main goal of GO-PCA signatures is to facilitate further analyses, the output of the method can also serve as a point of reference, since it does not depend on a multitude of more or less subjective decisions regarding the appropriate number of clusters, the importance of individual genes in each cluster, their functional interpretation, etc. Second, the use of GO terms facilitates comparisons across datasets, e.g., with immune cell data, as presented here. This could be especially relevant in trying to understand the expression contributions of the tumor microenvironment, e.g. from immune cells [44]. Third, GO-PCA is not a clustering algorithm, and provides a means for visualizing and interpreting the data without requiring samples to be assigned into discrete groups. In the analysis presented here, signatures from both the proliferation and the extracellular matrix (ECM) group of signatures were associated with the first principal components, yet they showed little to no correlation. It would therefore be difficult to decide whether to group samples primarily based on their ECM or their proliferation expression signature. In the GO-PCA signature matrix, “independent” signatures (i.e., signatures that exhibit no strong correlation or anti-correlation) are displayed side-by-side, which enables a multidimensional characterization of individual samples. Clustering can still be performed as a follow-up analysis, but it is not a precondition for obtaining an interpretable view of the data.

### Limitations and future work

In this work, all data analyzed were generated using microarrays. However, I have good reason to believe that in principle, GO-PCA will also perform well for data generated using different platforms (e.g., RNA-Seq). GO-PCA relies on two nonparametric methodologies (PCA and GO enrichment using the XL-mHG test) and therefore avoids strong assumptions about distributional properties of the data. Thus, generally speaking, GO-PCA will be applicable whenever PCA can be expected to recover meaningful axes of variation. It is important to note though that microarray and RNA-Seq data exhibit distinct technical biases [45], and might therefore benefit from different preprocessing procedures before PCA is applied. While GO-PCA was designed to require as little parameter tuning as possible, a “parameter” that currently requires adjustment on a case-by-case basis is the number and identity of genes included in the analysis. In order to avoid biasing the discovery of enriched GO term towards terms generally overrepresented among expressed genes, it is advisable to exclude genes thought not to be expressed from the analysis. In the current work, this was achieved by conservative application of a variance filter, a functionality which was directly built into GO-PCA. See S1 Text for a more in-depth discussion of this point and other GO-PCA parameters.

GO-PCA’s performance obviously depends on the quality of the gene annotations available. While this is true of any annotation-driven approach, it is currently unclear how well currently available GO annotations perform in capturing biological differences that are different or more subtle than the ones discovered for the datasets analyzed in this work. This issue has inspired a natural generalization of GO-PCA, where instead of relying on GO annotations, researchers can specify an arbitrary list of gene sets, which can represent functional units deemed more relevant for the dataset analyzed. GO-PCA was designed to accommodate this idea, by reading GO annotations (which can simply be represented by gene sets) from a plain-text file that can easily be modified to include different gene sets. In this way, “Gene Set-PCA” (GS-PCA), i.e., GO-PCA with arbitrary gene sets instead of GO annotations, can be applied even more generally.

Currently, the number of PCs tested by PCA is determined using a permutation-based strategy. Future research could be directed at also incorporating an algorithm that determines the number of genes significantly associated with each PC. It has been suggested to assess the association between individual variables (genes) and principal components using a “jackstraw” approach [46]. In combination with an FDR criterion [47], a jackstraw-like approach could allow GO-PCA to adjust the XL-mHG *L* parameter in a “smart” way for each PC. This could further improve GO-PCA’s statistical power in generating signatures for subtle signals of heterogeneity.

On an Intel Xeon E5620 CPU, it took GO-PCA between 57 and 126 seconds, and roughly 1 GB of memory, to generate the signatures for the three datasets analyzed here. This relatively short analysis time allowed bootstrap analysis to be performed without any parallelization. However, since the GO enrichment analysis for each PC is performed independently, parallelization could provide significant runtime improvements (at the expense of a larger memory footprint). In conclusion, GO-PCA represents a powerful and versatile method for the exploration of gene expression data, and demonstrates the potential of unsupervised algorithms that incorporate prior knowledge.

## Methods

### Obtaining a list of all protein-coding genes for human and mouse

Human and mouse Ensembl 79 genome annotations (.gtf files) were downloaded from ftp://ftp.ensembl.org and filtered for entries with feature name “gene”, and gene_biotype attribute “protein_coding” or “polymorphic_pseudogene”. The set of all associated “gene_name” attribute values was taken as the list of all protein-coding genes, yielding 20,114 genes. This resulted in a list of 19,742 genes for human, and a list of 22,007 genes for mouse.

### Obtaining the GO ontology and GO term-gene associations for human and mouse

The Gene Ontology structure (go-basic.obo) was downloaded from http://geneontology.org, with version “releases/2015-05-25” (http://viewvc.geneontology.org/viewvc/GO-SVN/ontology-releases/2015-05-25/go-basic.obo?revision=26059). UniProt Gene Ontology Annotation gene association files for human and mouse were downloaded from ftp://ftp.ebi.ac.uk/pub/databases/GO/goa/, both with versions generated on 2015-05-26. All annotations were propagated up the GO graph based on the “is_a” relationships (i.e., a gene that is annotated with a particular term was also considered annotated with all parent terms, since those represent more general categories). For GO terms in the “cellular component” domain, “part_of” relationships were treated the same as “is_a” relationships. Only annotations with evidence codes *IDA*, *IGI*, *IMP*, *ISO*, *ISS*, *IC*, *NAS*, or *TAS* were considered, on account of them representing manually curated annotations. This resulted in the exclusion of 55.4% and 32.5% of all GO annotations for human and mouse, respectively. I further removed GO terms that were either too broad (defined as having more than 200 genes annotated with them), or too specific (defined as having less than 5 genes annotated with them). Finally, I identified all instances where two or more GO terms had identical sets of genes annotated with them. For example, the term “organellar large ribosomal subunit” (GO:0000315) had the same 15 genes annotated with it as its child term, “mitochondrial large ribosomal subunit” (GO:0005762). In cases like this, where a parent term had identical sets of annotated genes as the child term, I removed the parent term, resulting in the exclusion of 582 and 555 terms for human and mouse, respectively. This resulted in a final set of 6,675 and 7,503 terms for human and mouse, respectively, which were neither too broad nor too specific, and not redundant with any related term.

### Principal component analysis (PCA)

PCA was performed using the decomposition.PCA module from the scikit-learn Python package [48], version 0.16.1 (http://scikit-learn.org).

### Determining the number of principal components to test

Since GO-PCA performs GO enrichment analysis on individual principal components (PCs), it is important to determine the appropriate number of PCs to test in this way. Intuitively, datasets with smaller sample sizes can be expected to exhibit fewer non-trivial PCs, i.e., PCs that do not mostly represent noise. Since PCs are sorted by the amount of variance explained (in decreasing order), we will generally retain the first *D* PCs. GO-PCA relies on a permutation test to determine *D*: First, the expression values for each gene in the original expression matrix are permuted (independently for each gene). Then, PCA is applied to this randomized matrix, and the fraction of variance explained by the first PC is calculated (i.e., the normalized value of the largest eigenvalue of the covariance matrix). This procedure is repeated 15 times. Then, the mean and sample standard deviation of the 15 values obtained can be used to compute z-scores for the fraction of variance explained for each PC of the actual (non-permuted) expression matrix. In the analyses presented here, GO-PCA was configured to test any PC with a z-score of 2.0 or larger. This approach was motivated by a recent single-cell expression study that showed that the distribution of eigenvalues from permuted expression matrices was similar to the known asymptotic Marchenko–Pastur distribution for eigenvalues of random matrices (see Fig 5E in [4]). It also represents a slightly more conservative version of a test that has been shown to produce relatively accurate estimates of the dimensionality of various simulated datasets (see “Avg-Rnd” in [49]).

### Nonparametric GO enrichment analysis using the minimum hypergeometric (mHG) test

Given a ranked list of protein-coding genes (where the ranking was defined by the loadings associated with a particular principal component), I tested for GO enrichment using a Cython [50] (http://cython.org) implementation of the minimum hypergeometric (mHG) statistic [23, 25]. The mHG statistic calculates a hypergeometric enrichment p-value (equivalent to Fisher’s exact test) for *all N possible cutoffs* in a ranked list of *N* binary variables, and then selects the cutoff associated with the best (lowest) p-value. Due to the many tests performed, this p-value cannot be taken at face value and is treated instead as an enrichment statistic (note that smaller values indicate stronger enrichment). I refer to this value as *s* ^mHG^. The mHG test then employs a dynamic programming algorithm [25] to calculate the probability *p* ^mHG^ of obtaining a statistic as small as or smaller than *s* ^mHG^, when given a random permutation of the ranked list. This algorithm has a time complexity of O(NK) (where *K* is the number of variables with value *TRUE*, or “1”), as opposed to the computationally infeasible O(N!) time complexity that would be required for explicitly enumerating all possible permutations. By definition, *p* ^mHG^ is the exact p-value associated with *s* ^mHG^.

I furthermore extended the mHG test by introducing two parameters, *X*, and *L*, and modified the dynamic programming algorithm used to calculate the mHG p-value *p* ^mHG^ to take these new parameters into account [24]. These parameters enable some control over which cutoffs are tested for enrichment, based on application-specific intuitions and requirements. The first parameter, *X*, ignores all cutoffs at which less than *X* positive (i.e., 1-valued) variables have been encountered. This criterion is designed to avoid situations where enrichment of only a very small subsets of all positive variables at the top of the ranked list is reported as significant. In GO-PCA, I adjust this parameter for each GO term, so that cutoffs which have a fraction of less than *X*_frac_ (or less than *X*_min_, whichever number is larger) of the genes annotated with that term above them are not tested for enrichment. This ensures that enrichment is based on a significant fraction of all genes annotated with the GO term, and serves to increase the likelihood of the signature labels representing the true underlying biological processes. In the analyses presented here, I used *X*_frac_ = 0.25 and *X*_min_ = 5. As an example of the effect of these parameters, suppose that 30 genes in the dataset are annotated with the GO term “regulation of cell cycle”. Then the first cutoff to be tested for enrichment needs to have at least ⌈0.25*30⌉ = 8 genes annotated with with this GO term located above it. The second parameter, *L*, limits the cutoffs tested to the first *L* ranks (*L* < *N*). This is designed to avoid cases where very weak enrichment (e.g. 1.5-fold) is reported as highly significant, solely because it is observed at a very low cutoff. For example, in a list of 10,000 genes, testing cutoffs of 5,000 and lower often makes no sense, as any biologically meaningful enrichment is expected to result from gene ranked much higher in the list. In my experience, any enrichment that can only be detected at such a low cutoff is likely the result of extrinsic biases and does not constitute a biologically meaningful signal. (This effect was also recognized as an important problem in the development of GSEA [14]). In GO-PCA, I use *L* ≈ *N*/8, due to the “two-sided” nature of the test (for a “one-sided” test, I suggest using *L* ≈ *N*/4).

Like the mHG test statistic, the XL-mHG statistic is calculated as the minimum hypergeometric p-value over a set of cutoffs (however, this set is restricted by *X* and *L*). Therefore, each individual XL-mHG test is associated with a cutoff k* at which this minimal p-value is achieved. For each GO term found to be significantly enriched, I define the set of genes “driving” the enrichment as those annotated with the GO term and located above the cutoff k* in the ranked list of genes.

### GO-PCA Part I: Finding and filtering of GO terms significantly enriched among genes ranked by their principal component loadings

The “backbone” of GO-PCA consists of a simple algorithm: *Step 1)* Determine the number of principal components to test *D* using the permutation test described above. *Step 2)* Perform PCA on the gene expression matrix (treating the genes as variables and the samples as observations), and extract the gene loadings associated with the first *D* principal components (PCs). *Step 3)* For each PC, rank genes by their loadings, in both descending and ascending order—this produces two gene rankings. Genes positioned at or near the top of either ranking contribute strongly to this PC, but in opposite directions: For a particular sample, strong expression of genes with very positive loadings result in a large PC score, while high expression of genes with very negative loadings result in a small PC score. *Step 4)* For each ranking, determine enriched GO terms using the XL-mHG test (see above), and apply a Bonferroni-corrected p-value threshold. For both human and mouse, I obtained *m* ≈ 10,000 GO terms (conservatively rounded up; see above). Therefore, for each PC, GO-PCA performs 2*m ≈ 20,000 tests. For a significance threshold of *α* = 0.05, the Bonferroni-corrected threshold would therefore be *α*_B_ = *α*/20,000 = 2.5*10^−6^. GO-PCA uses an even more conservative threshold of *α*_B_ = 1*10^−6^. However, GO-PCA does not perform any adjustment for the number of PCs tested. I have found that, maybe due to the conservative nature of *α*_B_, this does not appear to result in a large number of obviously meaningless signatures being generated, at least for the number of PCs tested here. Not adjusting *α*_B_ by the number of PCs also has the advantage that the same signatures are generated by the first principal components when GO-PCA is re-run with *D*′ ≠ *D*. *Step 5)* Generate a signature based on each enriched GO term (see below).

Along with this “backbone”, GO-PCA also filters the enriched GO terms to mitigate redundancies that result in part from the nested structure of the gene ontology: The set of genes annotated with a “child” GO term (representing a more specific functional category) often overlaps substantially with the set of genes annotated with its parent term, since the parent term “inherits” the annotations of all of its children. Therefore, a “local” filter is applied *independently for each PC and each gene ranking* (i.e., it is applied separately to the GO terms found to be enriched in each ranking). The key intuition that I used to devise the local filter is that among all the GO terms found to be significantly enriched for a specific PC and ranking, some GO terms are more strongly enriched than others, and those GO terms likely provide a more accurate description of the biological process underlying the observed expression pattern. To quantify the strength of enrichment for each GO term found to be significantly enriched (i.e., the *effect size* associated with the enrichment, as opposed to its significance), I developed an enrichment score for the XL-mHG test [24]. GO terms are ranked by this enrichment score (in descending order), and GO terms with lower scores are tested for whether their enrichment is still significant enriched (as judged by the XL-mHG p-value) when the genes driving the enrichment of the more enriched GO terms are removed from the analysis. Each signature that failed this test was considered redundant and removed from the list of enriched GO terms. More precisely, I applied the following filtering procedure: *Step 1)* Rank enriched GO terms by their fold enrichment (in descending order), and initialize a set of “seen” genes with the genes in the signature derived from the first GO term. *Step 2)* Remove all “seen” genes from the data and re-test the enrichment of the second GO term using the XL-mHG algorithm. If the test is still significant (using the same p-value threshold *α*_B_ as before), keep the signature derived from the second GO term and add its genes to the set of “seen” genes. If the test is not significant anymore, discard the signature associated with the second GO term. *Step 3)* Repeat step 2 for the third GO term, then the fourth, and so on, until all enriched GO terms have been tested for redundancy. In summary, the local filter removes redundant GO terms resulting from the nested GO structure and the frequent association of genes with multiple terms. However, when presented with “sufficient evidence”, it allows GO terms from the same PC to share a significant number of genes.

While the previously described filter helps avoid redundancies within each principal component, I also found that strong biological effects (e.g., differences in the expression of cell cycle genes) are sometimes associated with multiple PCs. To mitigate these cross-PC redundancies, I also applied a second “global” filter that removes a signature generated by a PC if its associated GO term (or one of its parents or children) was previously used to generate a signature for another PC. (Note that this implies that enrichments associated with earlier PCs are prioritized over enrichments associated with later PCs, motivated by the fact that earlier PCs capture larger fraction of the total variance).

### GO-PCA Part II: Using significantly enriched and filtered GO terms to generate signatures

For each significantly enriched GO term that passes both the local and the global filter, GO-PCA generates a single signature using the following two-step procedure: First, all genes driving the enrichment (as defined above) are identified, and their average standardized expression is computed. Then the *X* genes whose expression is most strongly correlated with this average expression profile are identified (where *X* is adjusted for each GO term, see above), and their average standardized expression profile is used as a signature “seed”. Then, any of the remaining genes is added if their correlation with the seed is at least *R* (by default, *R* = 0.5). Compared to no filtering (i.e., *R* = −1.0), this results in signatures with more strongly correlated genes (see S11 Fig). The signature expression profile is then calculated as the average standardized expression of all genes in the signature.

### Analysis of human hematopoietic expression data (DMAP)

I downloaded the hematopoietic dataset generated by Novershtern et al. [26], including sample annotations, from http://www.broadinstitute.org/dmap/home. This data is fully processed, and contains expression levels for 8,968 genes that were expressed in the majority of samples from at least one cell type (see [26] for details). Each row in the data contains two gene identifiers: One Entrez ID and one gene symbol. Since a relatively large number of gene symbols (1,486, or approx. 17%) did not match the name of any protein-coding gene in my list (see above), I instead mapped the Entrez ID to gene symbols myself, using data from NCBI’s gene2accession file (ftp://ftp.ncbi.nlm.nih.gov/gene/DATA/gene2accession.gz, downloaded on 5/26/2015); Column 2 of that file contains Entrez IDs, and column 16 the corresponding gene symbols. This resulted in the exclusion of only 440 rows (4.9%), either because the Entrez ID was not found in the gene2accession file, or because the gene symbol the Entrez ID mapped to was not contained in my list of protein-coding genes. This resulted in an expression matrix containing 8,528 genes. For visualization and analysis, I used the annotations provided in the file DMap sample info.022011.xls to sort samples based on lineage (column 6). For the XL-mHG algorithm (see above), I set *L* = 1,000 and used default values otherwise. Signatures were sorted using the leaf ordering of a dendrogram generated by hierarchical clustering with Pearson correlation as the distance metric and using average linkage, as implemented in scipy’s clustering.hierarchy.linkage function.

### Analysis of mouse immunological expression data (IGP1)

Raw data for 681 samples comprising the Phase 1 dataset from the Immunological Genome Project (IGP) Consortium [36] were downloaded from NCBI GEO, acession number GSE15907 (http://www.ncbi.nlm.nih.gov/geo/query/acc.cgi?acc=GSE15907; all data were generated using Affymetrix GeneChip Mouse Gene 1.0 ST microarrays.) I filtered for samples that were contained in Supplementary Table 1 of [36] (henceforth referred to as “IGP Annotation Table”), which resulted in a list of 650 samples. Raw data (CEL files) for these samples were processed using the rma function from the oligo R package [51], with the normalization parameter set to TRUE, resulting in the output being quantile-normalized. Probset IDs were mapped to gene symbols using mappings provided in the mogene10sttranscriptcluster.db R package, which relied on Entrez data from 3/17/2015, according to the package documentation. Following the methodology described in [36], in cases where multiple probesets mapped to the same gene symbol, the probset with highest average expression was used. (However, this affected less than 5% of genes for which data was available). The final number of mouse protein-coding genes which had at least one probeset mapped to them was 18,889 (85.8%). Replicates were grouped together according to their value in the “Sample class” column from the IGP Annotation Table, resulting in 214 groups containing between 2 and 7 replicates. For each group, the median expression values of all genes were calculated, resulting in an expression matrix with 214 columns, with each column corresponding to one “sample class”, referred to as “cell type” in this paper. Importantly, the panel also contained data for 10 stromal cell types, which represent an outgroup of non-hematopoietic cells unrelated to the other cell types in the data. Note that Jojic et al. sometimes obtained the same cell type from multiples tissues, e.g., CD+ dendritic cells from spleen and lymph nodes. In the analysis presented here, these were treated as separate “cell types”. Therefore, the number of “unique” cell types in the data is lower than 214.

As a validation of my processing pipeline, I reproduced Fig 2 from [36] (see S12 Fig). I then filtered the expression matrix to retain only the 8,000 most variable genes, in order to avoid biases resulting from the fact that only a subset of genes in the human genome are likely expressed in immune cells (see S1 Text) for further discussion of this point). GO-PCA was then run with the default parameter settings. Signatures were sorted using hierarchical clustering, as in the analysis of DMAP. For visualization purposes, samples were grouped according to their value in the “Sample group 2” column from the IGP Annotation Table. These values define what is referred to as “lineages” in this paper.

### Bootstrap analysis for assessing signature robustness

A bootstrap version of GO-PCA was implemented as part of the GO-PCA software (in the script bootstrap-go-pca.py). Using this script, 50 datasets were sampled with replacement from the original data, and GO-PCA was applied independently to each dataset (using the same parameters as in the analysis of the original dataset). The result was then used to visualize signature robustness, either as a summary statistic (see e.g., Fig 3a) or for each signature separately (see e.g., Fig 3b).

### Analysis of human glioblastoma expression data (GBM)

I curated a set of 479 glioblastoma transcriptomes from TCGA based on clinical annotation data from Supplementary Table 7 (“Clinical and Molecular Subclass Data Table”) in [40]. I first excluded patients that did not present with primary GBM (i.e., I excluded patients if they did not have “NO” in Column 2, “Secondary or Recurrant”), retaining 516 patients. I then downloaded annotation data for all GBM datasets in the TCGA data freeze from Oct 10, 2012 (data.freeze.txt from https://tcga-data.nci.nih.gov/docs/publications/gbm_2013/), and filtered for rows with column 5/“DATATYPE” equal to “Expression-Gene” and column 8/“DATA_LEVEL” equal to “3”). I then used these additional annotations to exclude samples that were annotated with any of the following terms (column 13, “ANNOTATION_CATEGORIES”): “item in special subset”, “normal class but appears diseased”, “qualified in error”. This resulted in the exclusion of 37 samples, and a final dataset of 479 samples. The expression data for these patients was extracted from the “Level 3” expression dataset (GBM.Gene Expression.Level 3.tar from https://tcga-data.nci.nih.gov/docs/publications/gbm_2013/). Of the 12,042 genes contained in these data, 1,257 (10.4%) were not contained in my list of human protein-coding genes (see above), and therefore excluded. The resulting matrix containing expression for 10,785 genes was quantile-normalized [52]. To obtain a set of expressed genes, I only retrained the top 6,000 most variable genes, removing 11.3% of the total variance in the data (see S1 Text for further discussion). I then ran GO-PCA with default parameters.

### Testing for association between GBM GO-PCA signatures and known GBM subtypes

To test whether signatures were associated with individual GBM subtypes (see Fig 6), I used the sample classifications provided in Supplementary Table 7 from [40]. For each signature, I compared signature expression in the two subtypes with the highest and second-highest median signature expression using a two-sided Mann-Whitney U test.

### Software

GO-PCA is free and open-source software and can be found at https://github.com/flo-compbio/gopca. Documentation is available at http://gopca.readthedocs.org. At the time of writing, this includes installation instructions for both Ubuntu Linux and Windows, and a demonstration of how to apply GO-PCA to the publicly available DMAP dataset analyzed in this paper.

The main analysis script is go-pca.py, and the overall workflow is outlined in S1 Fig. A discussion of important GO-PCA parameters is provided in S1 Text. GO-PCA provides various visualization and data processing options that were used in the generation of most of the figures in this manuscript (as indicated in figure legends; see also S2 Text for two additional visualization types available). Interested researchers are welcome to request features or directly contribute code through GitHub.

## Supporting Information

S1 Fig Overview of the GO-PCA workflow. **a** In a pre-processing step, a GO annotation file, containing a list of selected GO terms and genes annotated with them, is generated. **b** GO-PCA is run and the result is stored in Python’s binary “pickle” format. **c** Analysis scripts take the result file as input, and serve to process and visualize the results in various ways.
(TIF)

S2 Fig Application of GO-PCA to 211 human transcriptomes representing diverse hematopoietic lineages (DMAP). Shown is a heat map of the signature matrix generated by GO-PCA. Signatures are ordered using hierarchical clustering with correlation distance and average linkage. Samples are ordered using hierarchical clustering with Euclidean distance and average linkage. This figure was generated using the GO-PCA script gopca_plot_signature_matrix.py.
(TIF)

S3 Fig Homogeneity of GO-PCA signatures generated for DMAP. Shown is a box plot of all pair-wise correlation coefficients among the genes within each signature. This figure was generated using the GO-PCA script gopca_plot_within_signature_correlations.py.
(TIF)

S4 Fig Detailed view of selected GO-PCA signatures generated for DMAP. **a—c** For each signature, the signature expression profile is shown at the top, and the expression profiles of the individual genes in the signature are shown below. Genes are sorted using hierarchical clustering with correlation distance and average linkage. These figures were generated using the GO-PCA script gopca_plot_signature.py.
(TIF)

S5 Fig Application of GO-PCA to 214 mouse transcriptomes representing diverse hematopoietic lineages (IGP1). Shown is a heat map of the signature matrix generated by GO-PCA, as in S2 Fig.
(TIF)

S6 Fig Diagnostics for the application of GO-PCA to the IGP1 dataset. Signature homogeneity, as in S3 Fig. **b** Signature robustness, as in Fig 3a. **c** Simulation of smaller sample sizes, as in Fig 3b.
(TIF)

S7 Fig Detailed view of selected GO-PCA signatures generated for IGP1. Plots as in S4 Fig.
(TIF)

S8 Fig Homogeneity of GO-PCA signatures generated for DMAP. Shown is a box plot of all pair-wise correlation coefficients among the genes within each signature, as in S3 Fig.
(TIF)

S9 Fig Analysis of the robustness of GBM GO-PCA signatures using bootstrapping. **a-c** Overall detection rates, dependency on sample size, and signature-specific robustness analysis, as in Fig 3.
(TIF)

S10 Fig Correlation structure of GO-PCA signatures generated for the GBM dataset. Shown is a heat map of pairwise signature correlation coefficients, with signatures ordered as in Fig 5. Colored boxes indicate the groups of signatures indicated by bars of the same color in Fig 5 (blue = neuronal, gray = proliferative, yellow = immunological, red = extracellular matrix (ECM)-related).
(TIF)

S11 Fig The effect of filtering signature genes by their correlation with a signature “seed”. **a** Shown is a scatterplot comparing the median within-signature correlation values of each DMAP signature with (*R* = 0.5) and without (*R* = −1.0) filtering. The point marked in red corresponds to the “autophagy” signature. **b,c** Signature plots of the “autophagy” signature with (**c**) and without (**b**) filtering.
(TIF)

S12 Fig Correlation between cell types from the IGP11 dataset. Shown is a heat map of pairwise sample correlation coefficients (calculated after centering each gene by substracting its median expression value), with cell types ordered by their lineage identities, as in Fig 2 from [36]. Black boxes indicate lineage groupings.
(TIF)

S1 Text A discussion of key GO-PCA parameters. (PDF)

S2 Text Additional ways of visualizing GO-PCA results. (PDF)

S3 Text The potential importance of ubiquitin ligases in reticulocyte development. (PDF)

S1 File GO-PCA signatures generated for the DMAP dataset. (XLSX)

S2 File GO-PCA signatures generated for the IGP1 dataset. (XLSX)

S3 File GO-PCA signatures generated for the GBM dataset. (XLSX)
