# Supplementary figures and images for: GO-PCA: An Unsupervised Method to Explore Gene Expression Data Using Prior Knowledge
Source: PLoS One. 2015 Nov 17;10(11):e0143196. doi: 10.1371/journal.pone.0143196 (PMC4648502; doi:10.1371/journal.pone.0143196)

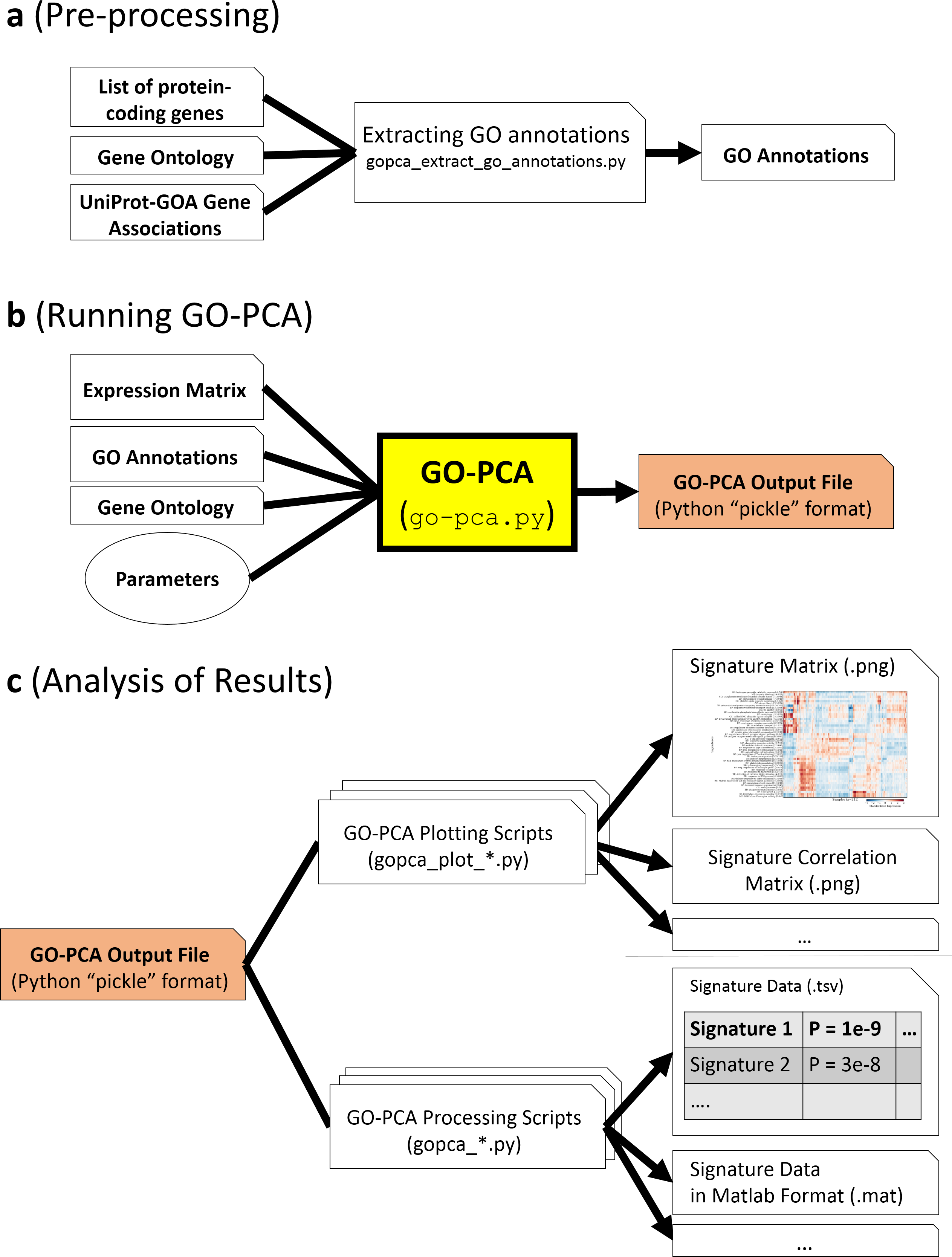

Supplement: S1 Fig — a In a pre-processing step, a GO annotation file, containing a list of selected GO terms and genes annotated with them, is generated. b GO-PCA is run and the result is stored in Python’s binary “pickle” format. c Analysis scripts take the result file as input, and serve to process and visualize the results in various ways. (TIF) [file pone.0143196.s001.tif]

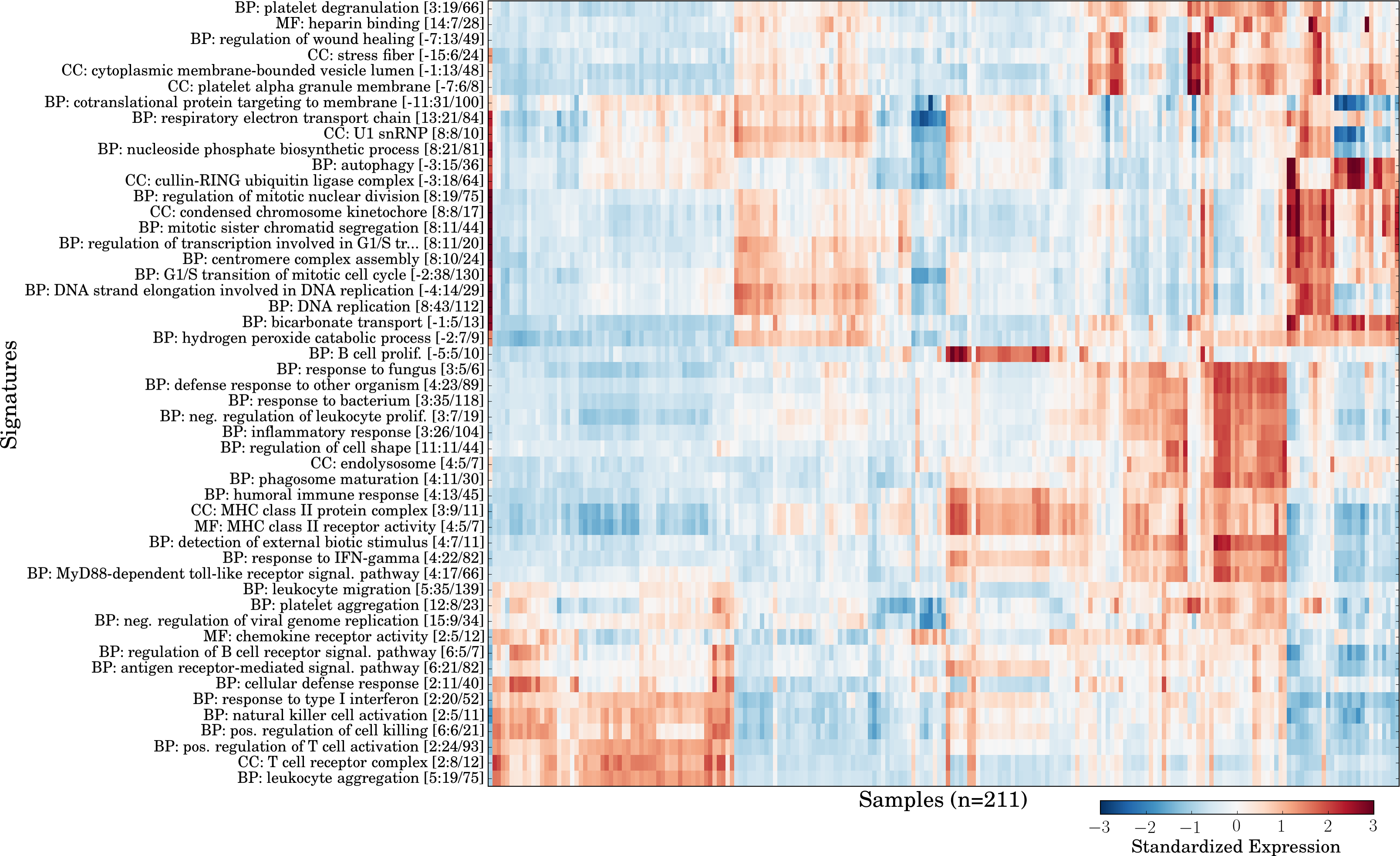

Supplement: S2 Fig — Shown is a heat map of the signature matrix generated by GO-PCA. Signatures are ordered using hierarchical clustering with correlation distance and average linkage. Samples are ordered using hierarchical clustering with Euclidean distance and average linkage. This figure was generated using the GO-PCA script gopca_plot_signature_matrix.py. (TIF) [file pone.0143196.s002.tif]

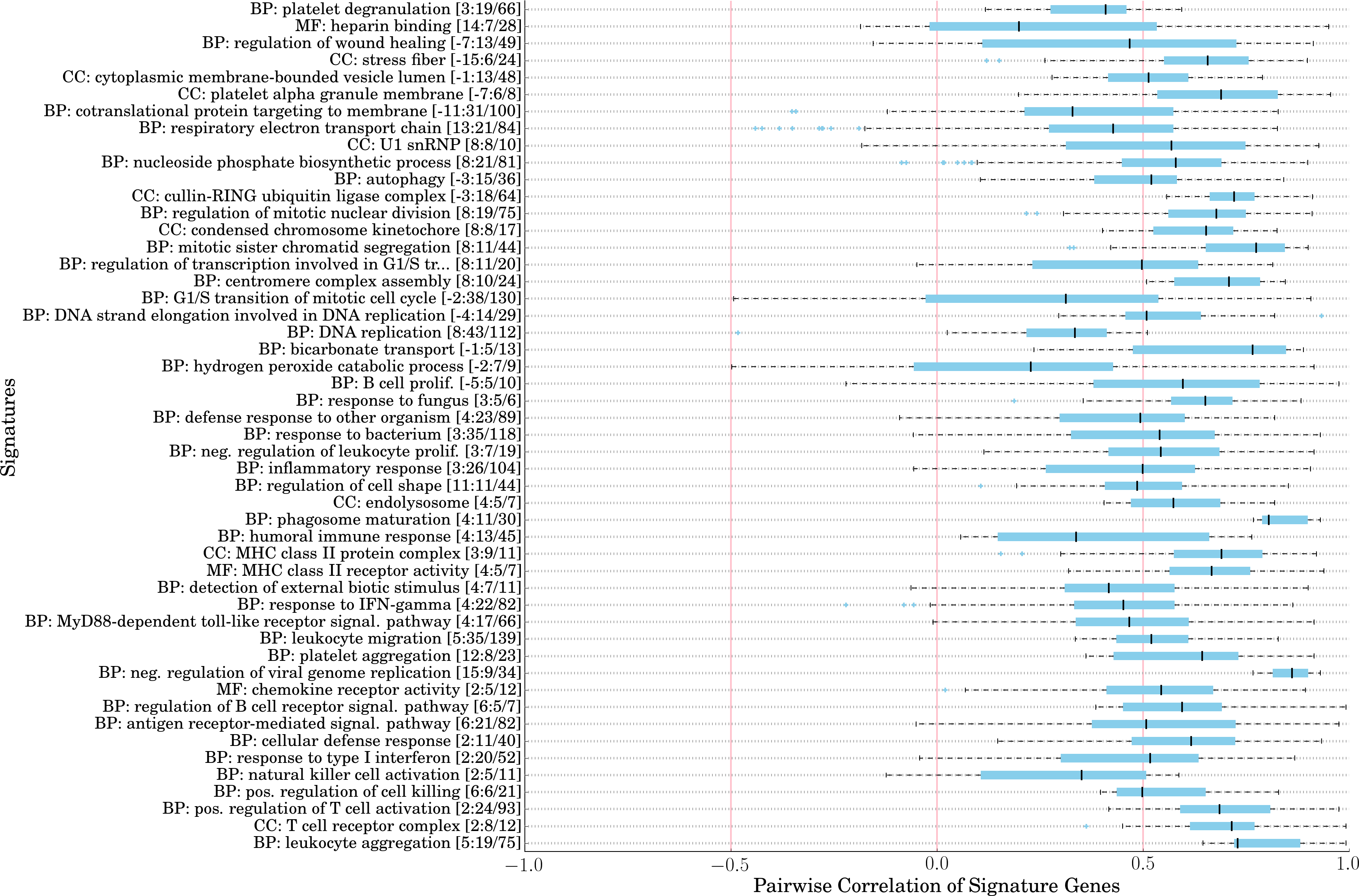

Supplement: S3 Fig — Shown is a box plot of all pair-wise correlation coefficients among the genes within each signature. This figure was generated using the GO-PCA script gopca_plot_within_signature_correlations.py. (TIF) [file pone.0143196.s003.tif]

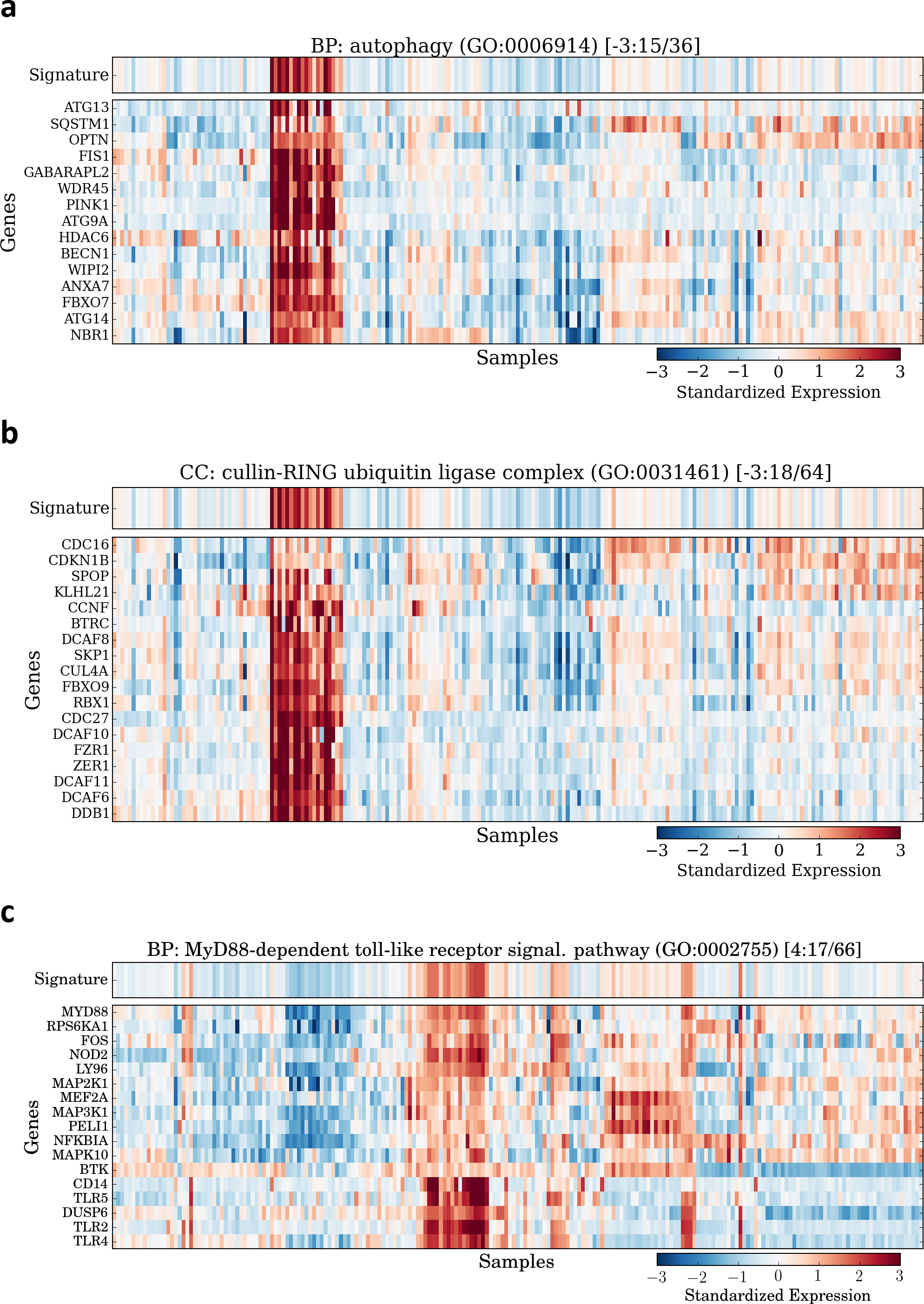

Supplement: S4 Fig — a—c For each signature, the signature expression profile is shown at the top, and the expression profiles of the individual genes in the signature are shown below. Genes are sorted using hierarchical clustering with correlation distance and average linkage. These figures were generated using the GO-PCA script gopca_plot_signature.py. (TIF) [file pone.0143196.s004.tif]

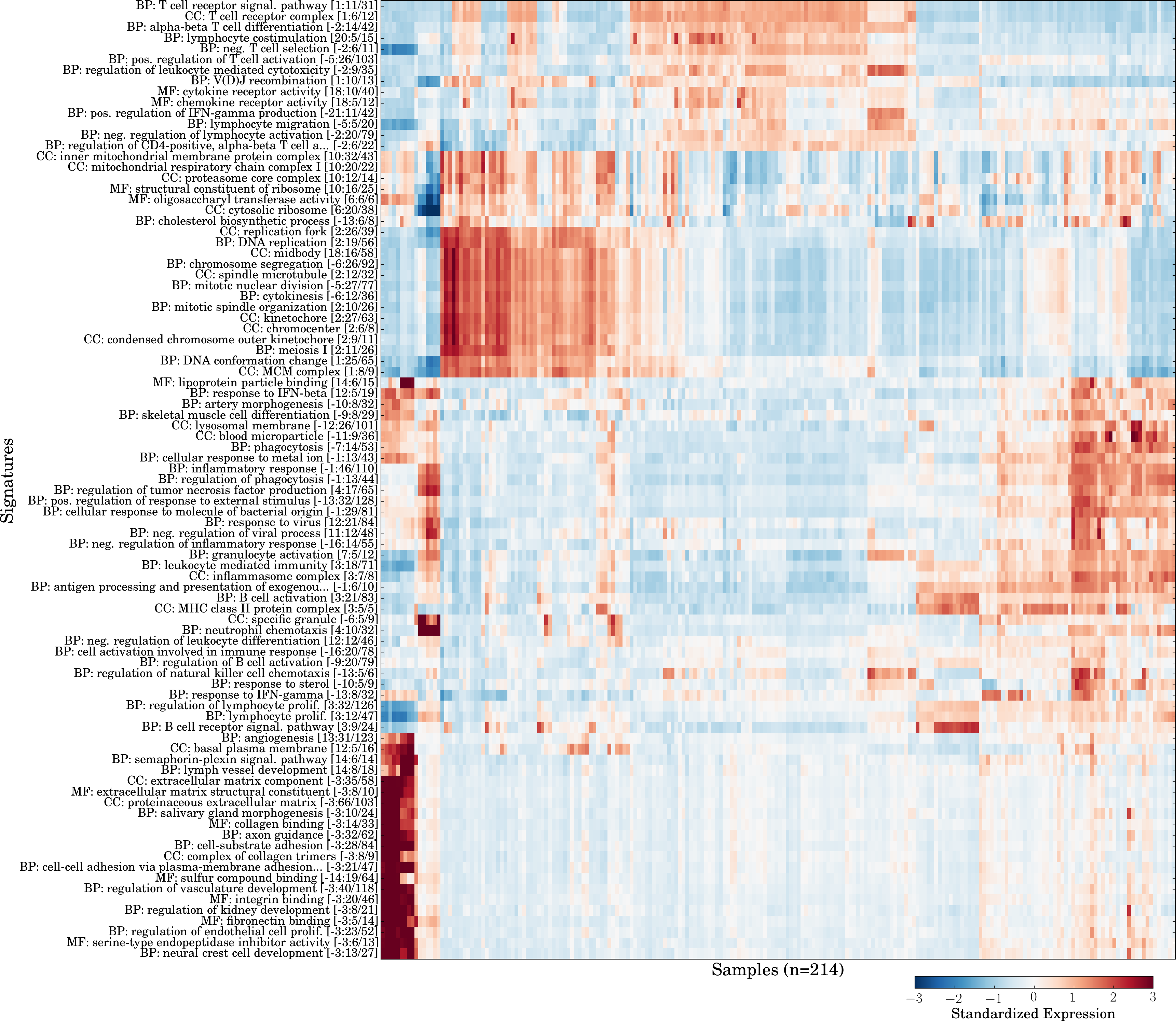

Supplement: S5 Fig — Shown is a heat map of the signature matrix generated by GO-PCA, as in S2 Fig. (TIF) [file pone.0143196.s005.tif]

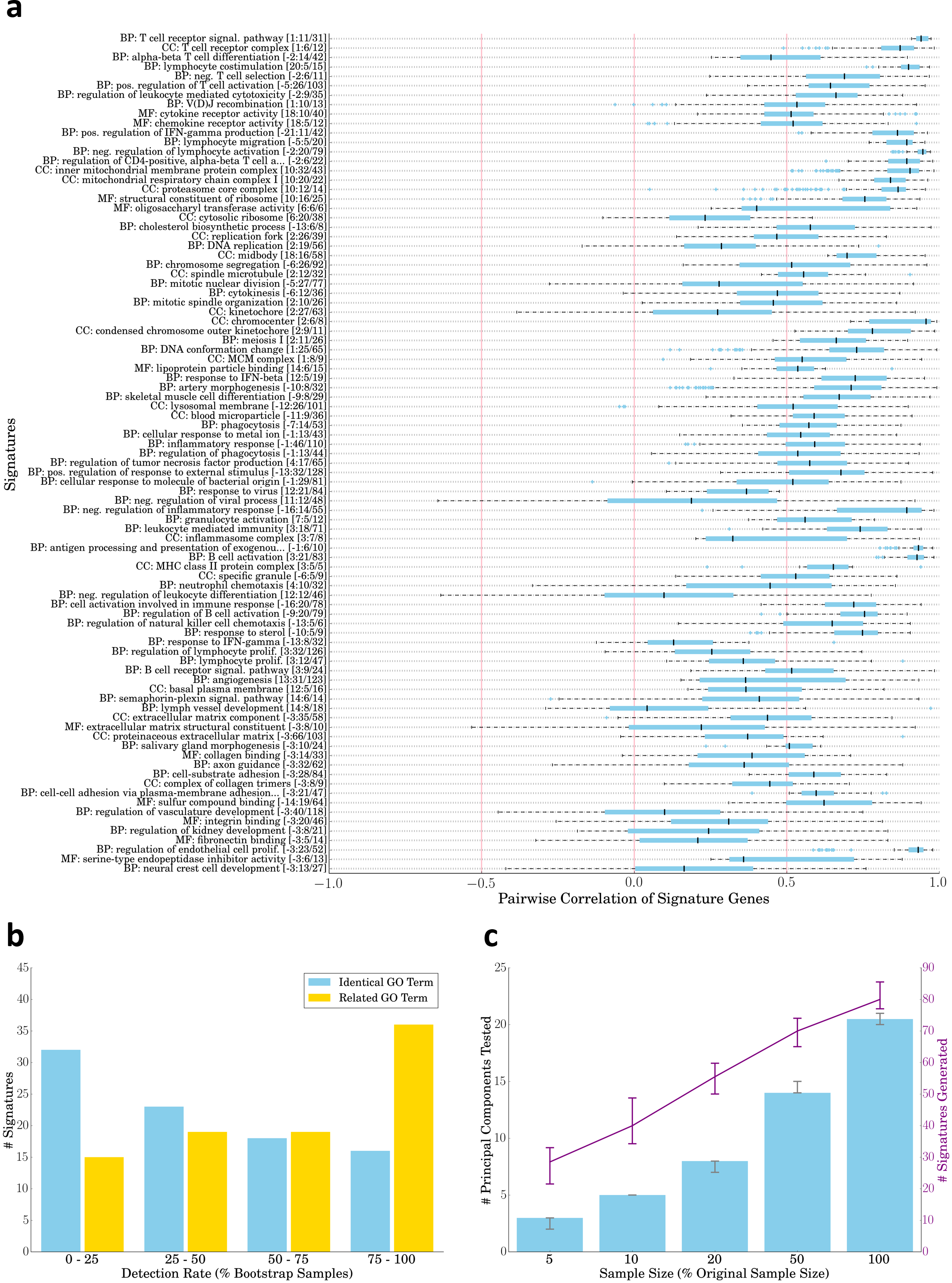

Supplement: S6 Fig — Signature homogeneity, as in S3 Fig. b Signature robustness, as in Fig 3a. c Simulation of smaller sample sizes, as in Fig 3b. (TIF) [file pone.0143196.s006.tif]

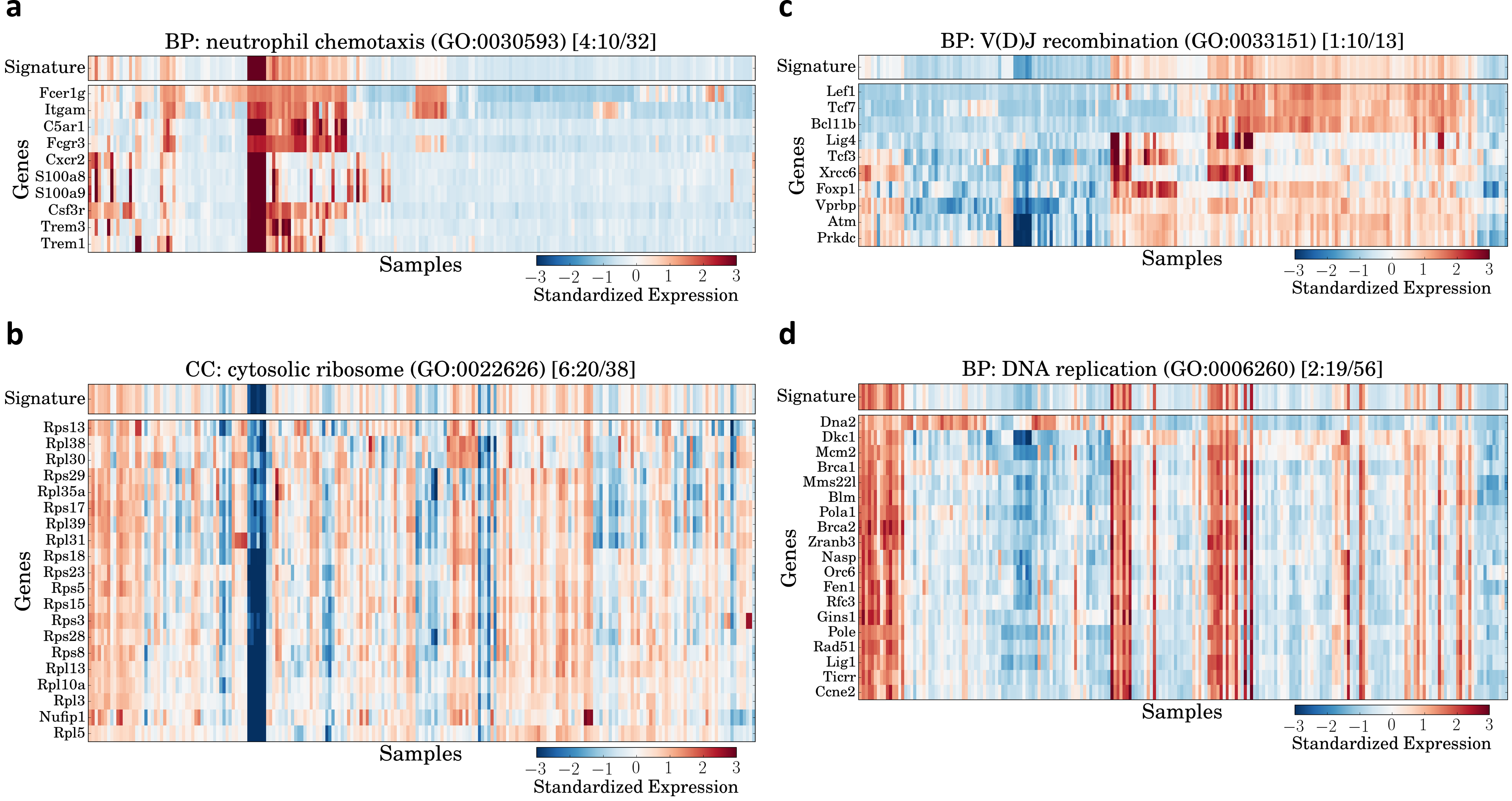

Supplement: S7 Fig — Plots as in S4 Fig. (TIF) [file pone.0143196.s007.tif]

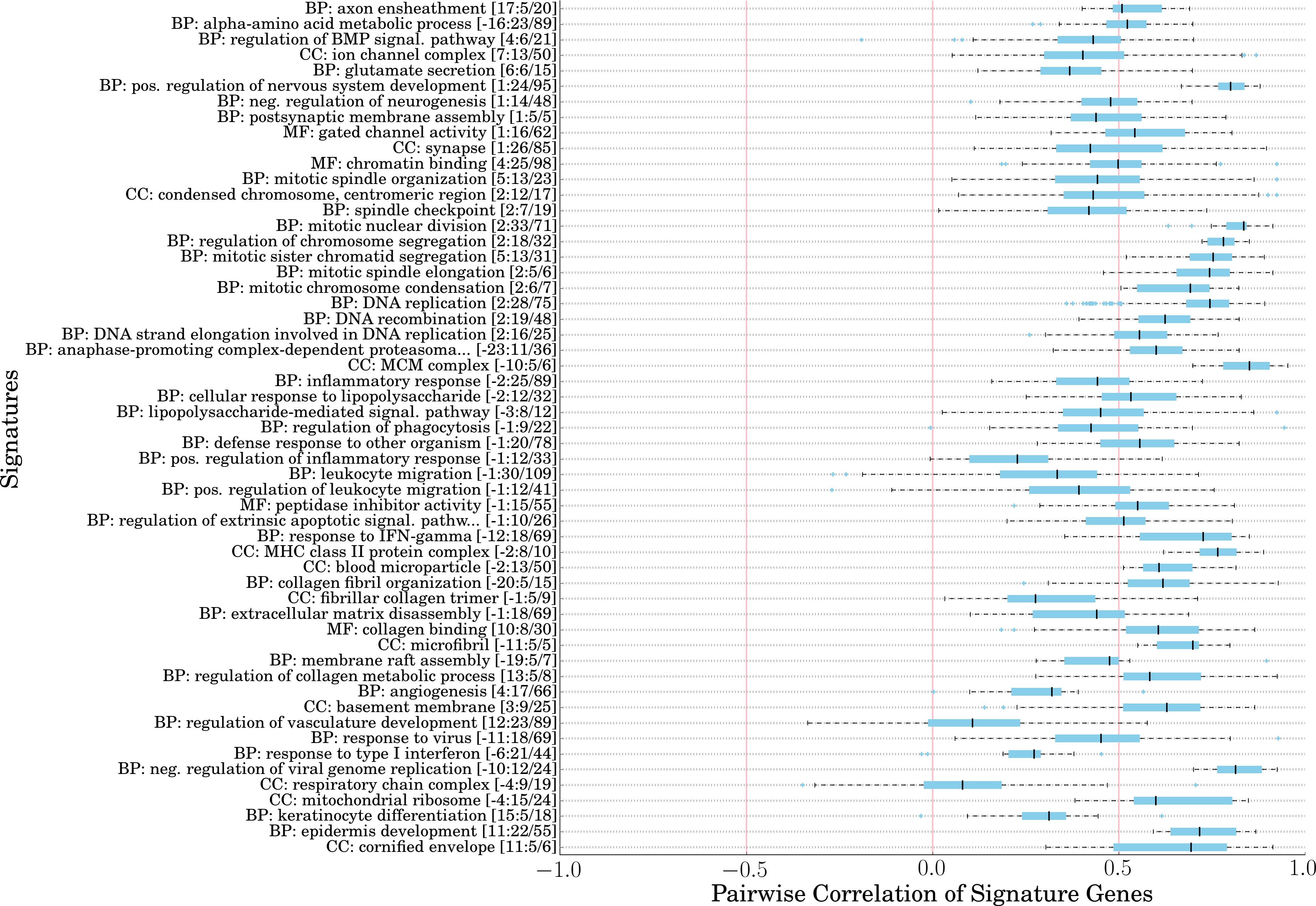

Supplement: S8 Fig — Shown is a box plot of all pair-wise correlation coefficients among the genes within each signature, as in S3 Fig. (TIF) [file pone.0143196.s008.tif]

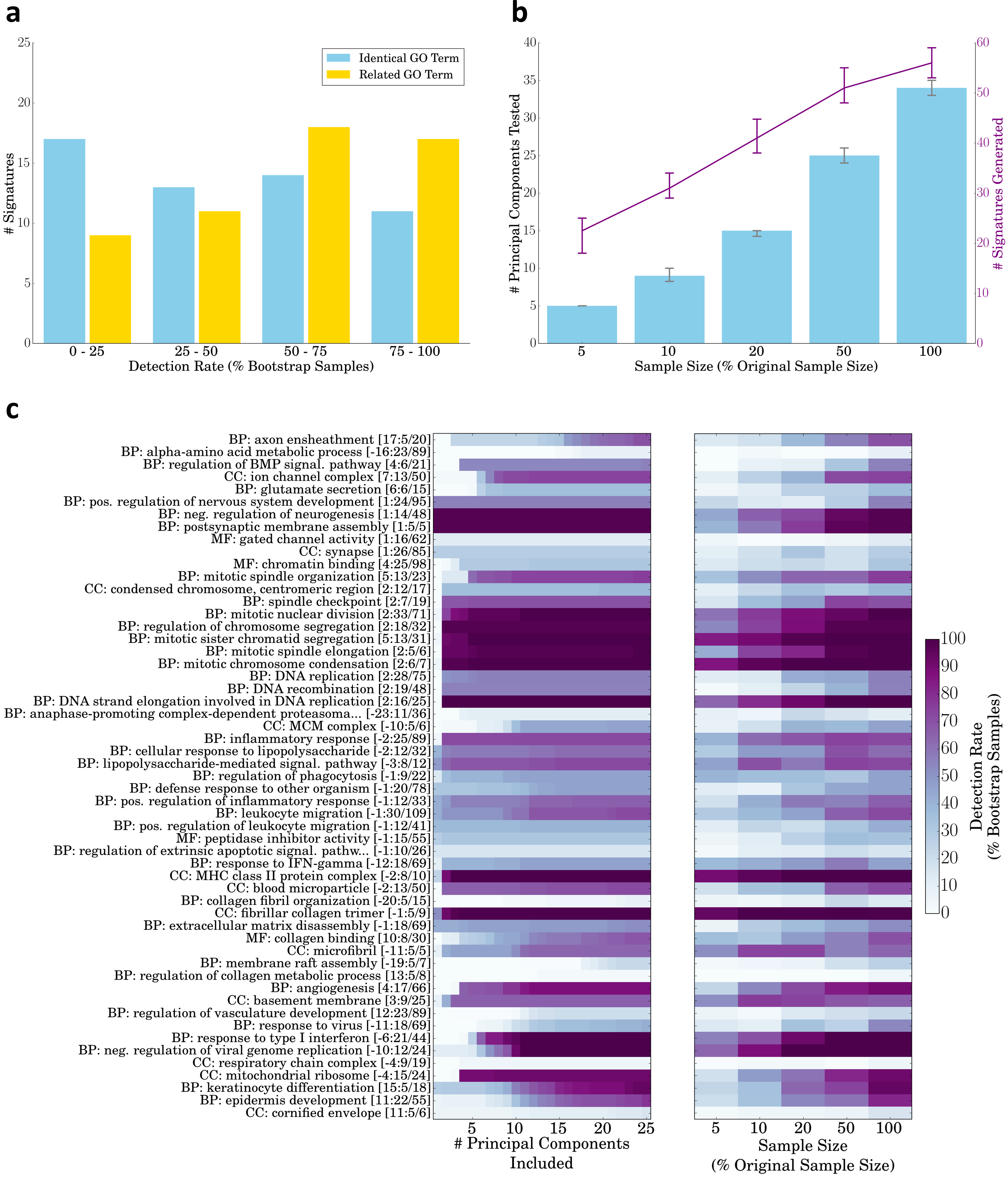

Supplement: S9 Fig — a-c Overall detection rates, dependency on sample size, and signature-specific robustness analysis, as in Fig 3. (TIF) [file pone.0143196.s009.tif]

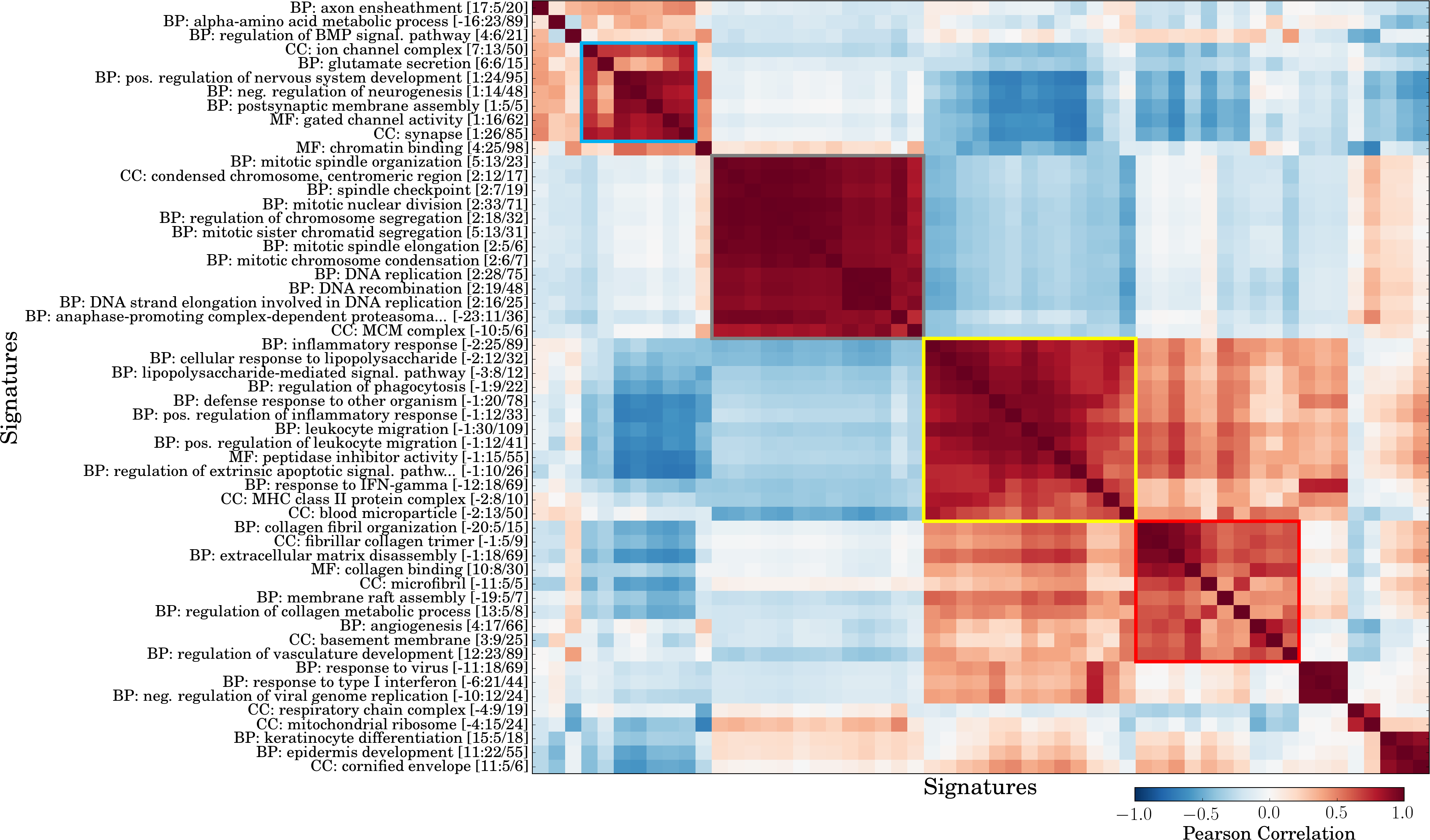

Supplement: S10 Fig — Shown is a heat map of pairwise signature correlation coefficients, with signatures ordered as in Fig 5. Colored boxes indicate the groups of signatures indicated by bars of the same color in Fig 5 (blue = neuronal, gray = proliferative, yellow = immunological, red = extracellular matrix (ECM)-related). (TIF) [file pone.0143196.s010.tif]

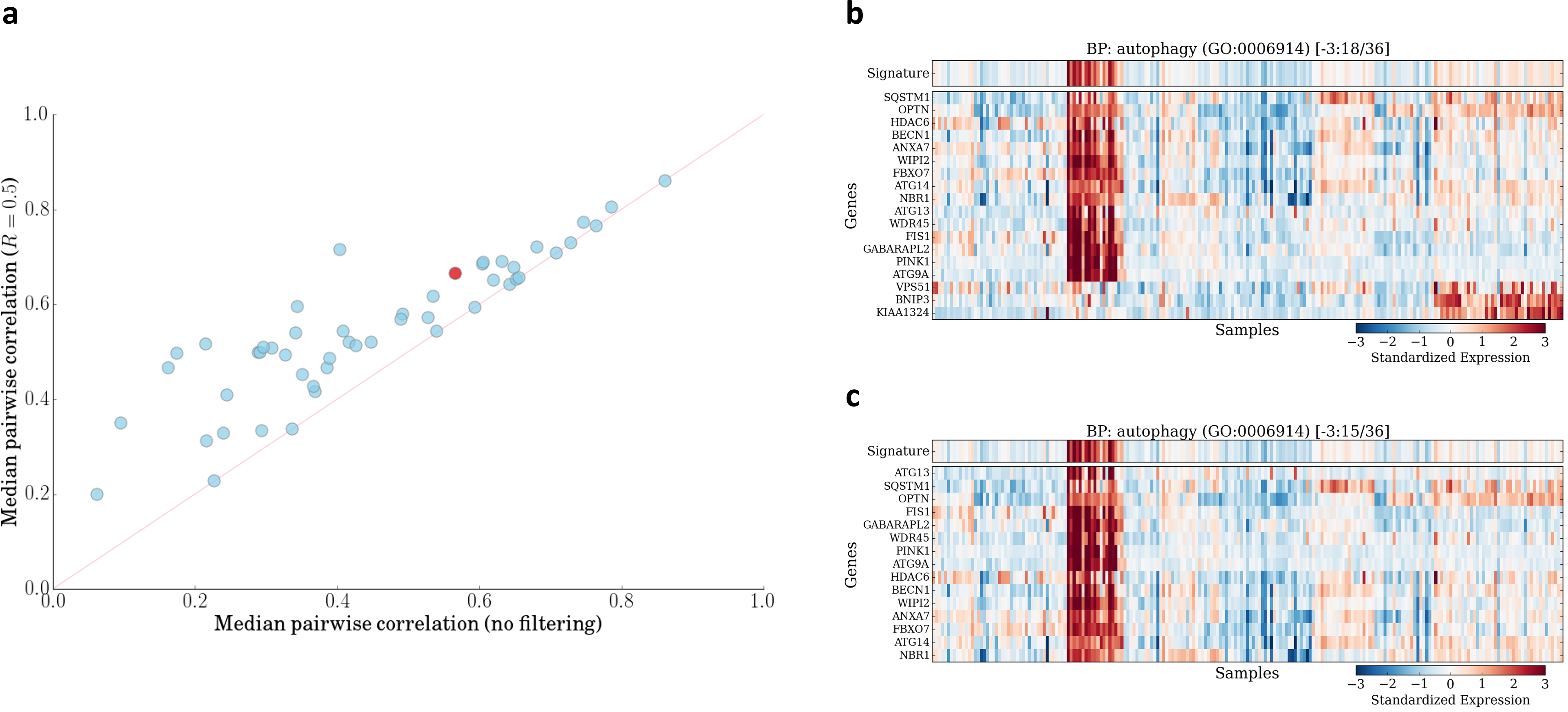

Supplement: S11 Fig — a Shown is a scatterplot comparing the median within-signature correlation values of each DMAP signature with (R = 0.5) and without (R = −1.0) filtering. The point marked in red corresponds to the “autophagy” signature. b,c Signature plots of the “autophagy” signature with (c) and without (b) filtering. (TIF) [file pone.0143196.s011.tif]

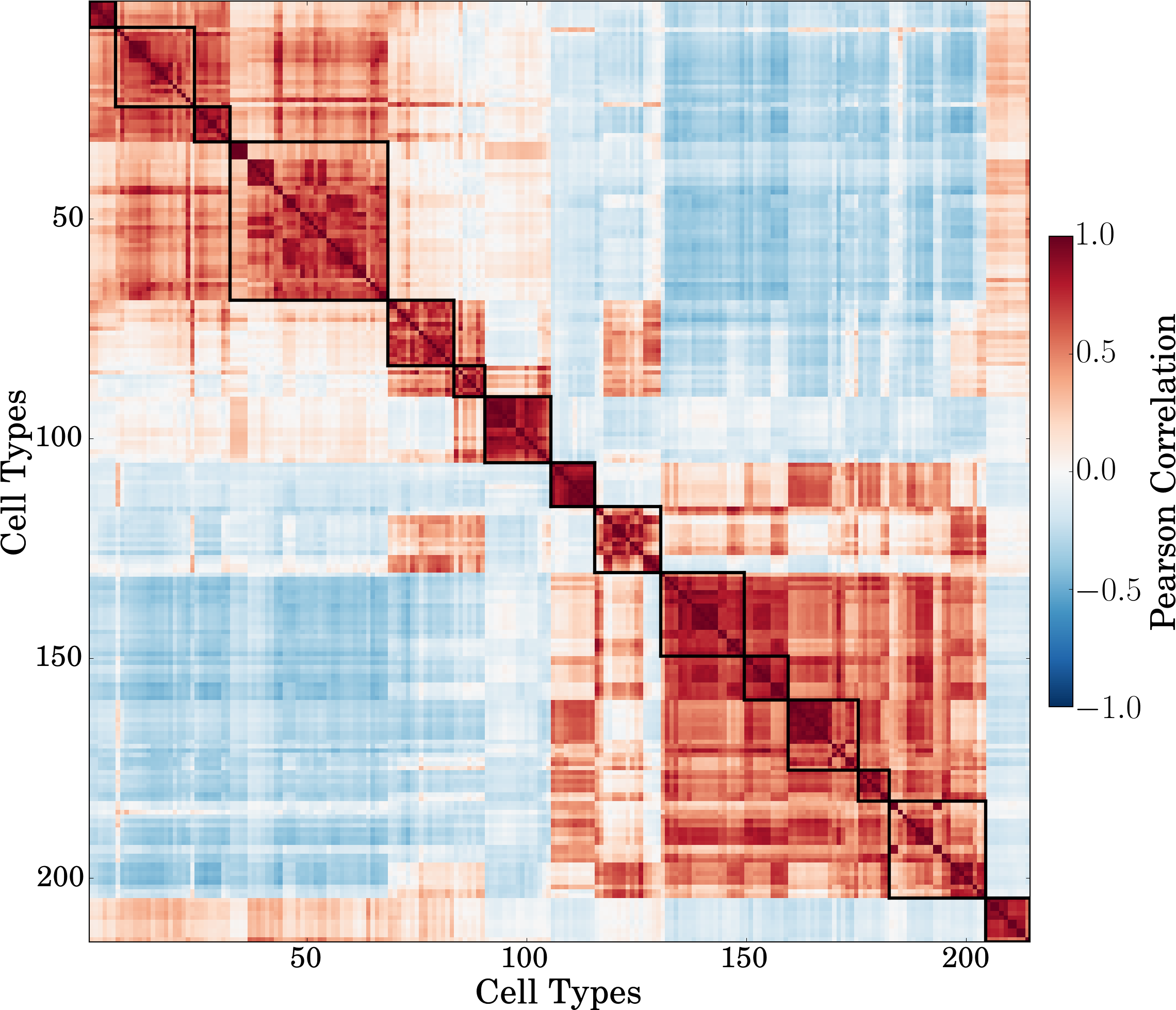

Supplement: S12 Fig — Shown is a heat map of pairwise sample correlation coefficients (calculated after centering each gene by substracting its median expression value), with cell types ordered by their lineage identities, as in Fig 2 from [36]. Black boxes indicate lineage groupings. (TIF) [file pone.0143196.s012.tif]
