# Supplementary material for: GO-PCA: An Unsupervised Method to Explore Gene Expression Data Using Prior Knowledge
Source: PLoS One. 2015 Nov 17;10(11):e0143196. doi: 10.1371/journal.pone.0143196 (PMC4648502; doi:10.1371/journal.pone.0143196)
Supplement: S1 Text — (PDF) [file pone.0143196.s013.pdf]

Supporting Text S1: A discussion of key GO-PCA parameters

Overview

One of the goals in designing the GO-PCA algorithm was to ensure that in most "standard" applications, GO-PCA would produce useful results with the default parameter settings. This is not completely unrealistic, since GO-PCA is built around two non-parametric methodologies, namely PCA and the XL-mHG enrichment test. Currently, the only "parameter" that needs to be considered on a case-by-case basis is the choice of how many (and which) genes to include in the analysis. In any multicellular organism, only a certain fraction of all protein-coding genes of that organism is expressed in any specific cell or tissue type. If one treats the entire set of protein-coding genes as the "universe" of genes, when in fact only some of them are actually expressed in the samples obtained, this can result in an unwanted bias when conducting GO enrichment analyses (the "enriched" terms could simply represent biological functions enriched among the expressed genes). Therefore, it is strongly recommended to only include "expressed" genes in the GO-PCA analysis.[1] At the time of writing, users of GO-PCA have two options for achieving this: Either they filter their expression matrix themselves to only contain genes they believe to be expressed, or they use GO-PCA's *G* parameter (specified using the command line option "-G"), to filter for the most variable genes, as explained below.

After making sure that the set of genes analyzed is similar to the set of expressed genes, the default settings of the remaining parameters (P, X_{frac}, X_{min}, L, R) should allow users to obtain a useful result, bearing in mind the general caveats of the method (see Discussion section in the main text). However, by understanding how these additional parameters impact the output of GO-PCA, users can adjust them to better suit their goals and the specific characteristics of the dataset analyzed.

G – Filtering the expression matrix to only include the G genes with the largest variance

While *G* only affects a pre-processing step that is not technically part of the GO-PCA algorithm, it can nevertheless have a strong and direct impact on the signatures generated by GO-PCA. Setting of this parameter directs GO-PCA to perform variance filtering on the expression matrix, retaining only the G genes with the largest sample variance. The application of this filter is recommended in most applications that involve microarray data, since otherwise a large proportion of the genes in the analysis consist of genes that are not actually expressed in the samples under study. A large number of unexpressed genes can result in artificially low p-values when GO-PCA performs the XL-mHG test for enrichment on the list of genes ranked by their PC loadings, since those genes will tend to have low absolute loadings (they are not correlated with any major axes of variation in the data). When p-values are artificially low, many GO terms will be found to be enriched that simply represent functional categories of genes that are generally expressed (as opposed to not expressed at all) in the samples under study. Signatures based on such biased GO enrichment results then are not expected to exhibit very interesting expression patterns, and do not describe interesting ways in which the samples differ. I therefore advocate a conservative filtering, e.g. taking $G = M/2$, where M is the number of genes represented on the array (equivalent to assuming that half of the measured genes are expressed). However, if *G* is reduced too much, the analysis might be hampered by two factors: First, the signal in some principal components might be lost, and second, the

---

[1] When an exact threshold for what constitutes "expression" is not available, it is better to err on the conservative side and underestimate the number of expressed genes. For the analyses presented in the main text, I estimated the number of expressed genes as approximately half of the genes represented on the microarray.

statistical power of the GO enrichment test might be too low at the specified p-value threshold (the default threshold is 10^{-6}, see below). *By default, no filtering is performed (G=0).*

For count-based expression methods such as RNA-Seq, a variance filter might not be optimal, and a threshold based on absolute expression levels (e.g., RPKM > 5.0) might be preferable. At the time of writing, GO-PCA does not supporting filtering based on absolute expression, and the user would have to perform this pre-processing step separately.

P – The p-value threshold used in the GO enrichment analyses

The value of this parameter obviously has a direct impact on the number and identity of the signatures generated, since only GO terms whose enrichment meets the specified significance threshold are used for generating signatures. **By default, *P* = 10^{-6}.** This threshold was chosen as a conservative threshold for testing the enrichment of all GO terms based on the loadings of a single PC (see discussion in the Methods section of the paper). However, since many PCs are tested for enrichment (i.e., easily more than 10 for large and heterogeneous datasets), selecting a conservative threshold is actually important in order to avoid too many false positive associations. Alternatively, the P-value could be adjusted for the number of PCs tested (e.g., based on the false discovery rate). However, I would argue that this would make the output of GO-PCA more difficult to interpret, and it would also introduce a complication whereby different signatures could be produced for the same PC, depending on how many other components are tested. Therefore, at the time of writing, GO-PCA's approach is to not apply a correction for the number of PCs tested.

X_{frac} and X_{min} – Determining what fraction of genes with a given function can form a "signature"

The mHG enrichment test that GO-PCA relies on (in a modified form) for detecting enriched GO terms can sometimes detect the enrichment of a very small subset of genes annotated with a specific GO term. For example, suppose that 100 genes in our expression matrix are annotated with the GO term "DNA replication", and suppose we observe very large loadings for a few of those genes (let's say 10) for a given PC. This might then result in a positive enrichment test. However, how willing are we to conclude that samples differ with respect to DNA replication activity, based on the behavior of only 10% of genes annotated with that term? After all, these 10 genes might also serve a different function that is not yet documented by any GO annotations. Therefore, to increase the probability of a signature label actually "meaning what it says", GO-PCA requires "enrichment" of a GO term to be based on at least a certain fraction X_{frac} of *all* genes annotated with that term (where "all" refers to the set of genes in the expression matrix, not all protein-coding genes of the organism). **By default, X_{frac} = 0.25.** However, since some terms have very few genes annotated to them 25% would correspond to only two or three genes. Since enrichment based on two or three genes is not very interesting, GO-PCA also requires a minimum *absolute* number of genes X_{min} to form the basis of enrichment. **By default, X_{min} = 5.** In summary, for each GO term, the *X* parameter of the XL-mHG enrichment test is calculated as $X = \max(X_{frac}*k, X_{min})$, where *k* is the number of genes in the dataset that are annotated with that term.

L – Determining where to look for enrichment

Besides X_{frac} and X_{min}, a third parameter influencing the enrichment test is *L*, which specifies which part of the ranked list of genes is tested for enrichment. Due to the statistical nature of the mHG test, GO terms can sometimes be detected as enriched based on the fact that there is a slight (say, 1.5-fold) enrichment of genes annotated with the GO term among, say, the top 50% of genes in the list. This can be highly statistically significant (as it is unlikely to arise purely by chance), but at the same time not very

biologically interesting (since we are looking for a small number of genes with very large PC loadings, not a large number of genes with slightly higher than average loadings). To prevent a positive result in those situations (which would then lead to the generation of uninteresting signatures), the XL-mHG provides an *L* parameter, which prevents testing of "low cutoffs". For example, if the number of genes in the data is *m*, setting *L=m/4* will result in only the top 25% of the list to be tested for enrichment. I have found this to be a reasonable choice for GO enrichment analysis in general. For GO-PCA, **by default, *L=m*/8** (since enrichment is tested for genes ranked based on their loadings in both ascending in descending order).

R – The correlation threshold used to determine signature "membership"

In order for signatures to consist of genes with similar expression profiles, GO-PCA performs some filtering when deciding which genes annotated with a certain GO term to include in the corresponding signature. More specifically, it first generates a "seed" signature consisting of the *X* genes with the highest correlation to the average expression profile of the genes that form the basis of the enrichment (see the previous paragraph for information on how *X* is calculated). If there are more than *X* of these genes, the remaining genes are added to the signature if the correlation coefficient between their expression and the seed signature is larger than *R*. **By default, *R* = 0.5.** The idea of this parameter is that increasing it leads to smaller signatures with genes that are more tightly correlated.

Further Reading

For more details on the mHG test, both generally and in the context of specific applications, see [1], [2]. For a purely technical discussion and a description of the XL-mHG extension, see [3].

References

[1] E. Eden, D. Lipson, S. Yogev, and Z. Yakhini, "Discovering motifs in ranked lists of DNA sequences," *PLoS Comput. Biol.*, vol. 3, no. 3, p. e39, Mar. 2007.

[2] E. Eden, R. Navon, I. Steinfeld, D. Lipson, and Z. Yakhini, "GOrilla: a tool for discovery and visualization of enriched GO terms in ranked gene lists," *BMC Bioinformatics*, vol. 10, p. 48, 2009.

[3] F. Wagner, "The XL-mHG Test For Enrichment: A Technical Report," *ArXiv150707905 Stat*, Jul. 2015.
