# Supplementary material for: GO-PCA: An Unsupervised Method to Explore Gene Expression Data Using Prior Knowledge
Source: PLoS One. 2015 Nov 17;10(11):e0143196. doi: 10.1371/journal.pone.0143196 (PMC4648502; doi:10.1371/journal.pone.0143196)
Supplement: S2 Text — (PDF) [file pone.0143196.s014.pdf]

Text S2: Additional ways of visualizing GO-PCA results

While GO-PCA's primary output consists of the signature matrix, it also supports multiple other ways of visualizing its results. These visualizations serve more specialized purposes, and can help gain further insight into various aspects of the data. Several of these visualizations were introduced in the main text. Here, I discuss additional visualization options using the DMAP dataset as an example. Each of the figures below is generated using a different plotting script, as indicated in the text (see Figure S1 for an overview of the GO-PCA workflow).

GO Term-by-PC matrix

The GO term-by-PC matrix contains the p-values obtained by GO-PCA when testing for associations of GO terms with principal components (PCs), for each GO term that was used to generate a signature, and for each PC tested. As described in the Methods section of the paper, these p-values are obtained by first ranking genes by their PC loadings, and then applying the XL-mHG test for enrichment. For each GO term and each PC, two tests are conducted, based on descending and ascending order of the gene loadings, respectively. The matrix contains the p-values obtained for each test in separate columns. Frequently, a particular GO term is found to be associated with multiple PCs, which is not evident from the signature matrix (due to GO-PCA's filtering rules, these associations do not result in the generation of additional signatures). A figure displaying this matrix as a heat map (**Figure 1**) can be generated using the script `gopca_plot_term_by_pc_matrix.py`. The user can specify a p-value threshold, such that p-values that are greater than the threshold are not shown. By default, this threshold is set to 0.0001. The number following each GO term name indicates the number of genes in the dataset annotated with this term. For each GO term, the PC used to generate the signature is indicated using a yellow "x" symbol. The ordering of GO terms follows that of the signature matrix. This visualization therefore provides a more direct view of which GO terms are associated with which component(s), and was originally proposed by Dr. Meromit Singer.

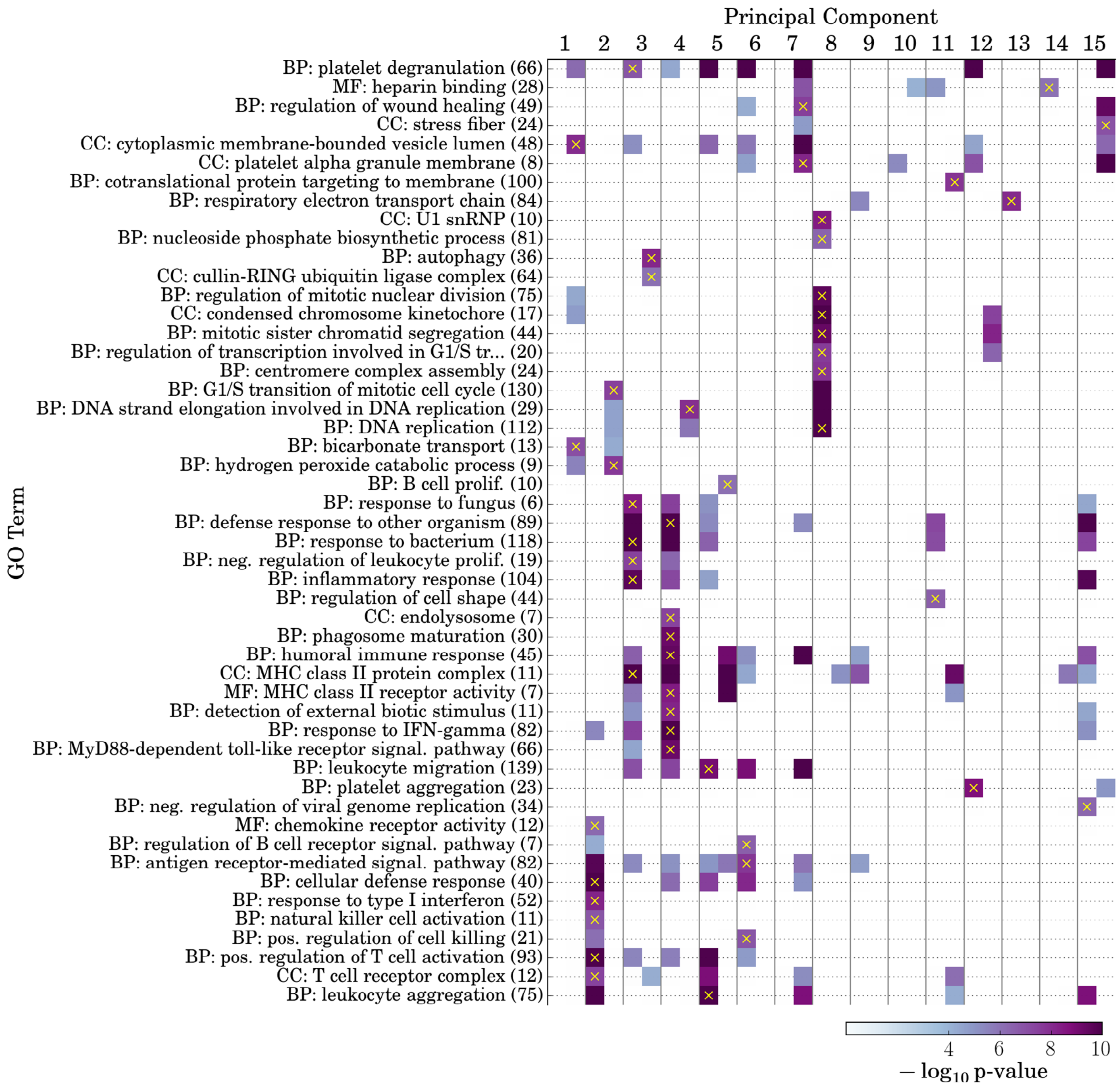

Figure 1: GO term-by-PC matrix. Heat map showing the significance of association between GO terms and principal components (PCs), as determined by the XL-mHG test, for the DMAP dataset. Only associations with p-values <0.0001 are shown. The GO terms shown are those used to generate signatures in the GO-PCA analysis of the DMAP dataset. Their ordering corresponds to the ordering of the signatures in the signature matrix (see Fig 2). Significant associations which led to the generation of a signature by GO-PCA are marked with a yellow “x”. For each PC, two columns are shown, corresponding to the sorting of genes by their loadings in descending and ascending order, respectively (note that the orientation of each PC is arbitrary).

Signature Principal Component Plot

This visualization consists of a plot of sample PC scores, with each sample represented by a circle filled with a color that represents the expression value of a specific signature in that sample, therefore providing a more detailed view of the relationship between "traditional" PC scores and signature expression. The four panels of (**Figure 2**) were generated using the script `gopca_plot_pc_scores.py`. This visualization was inspired by comments from the anonymous reviewer of the manuscript.

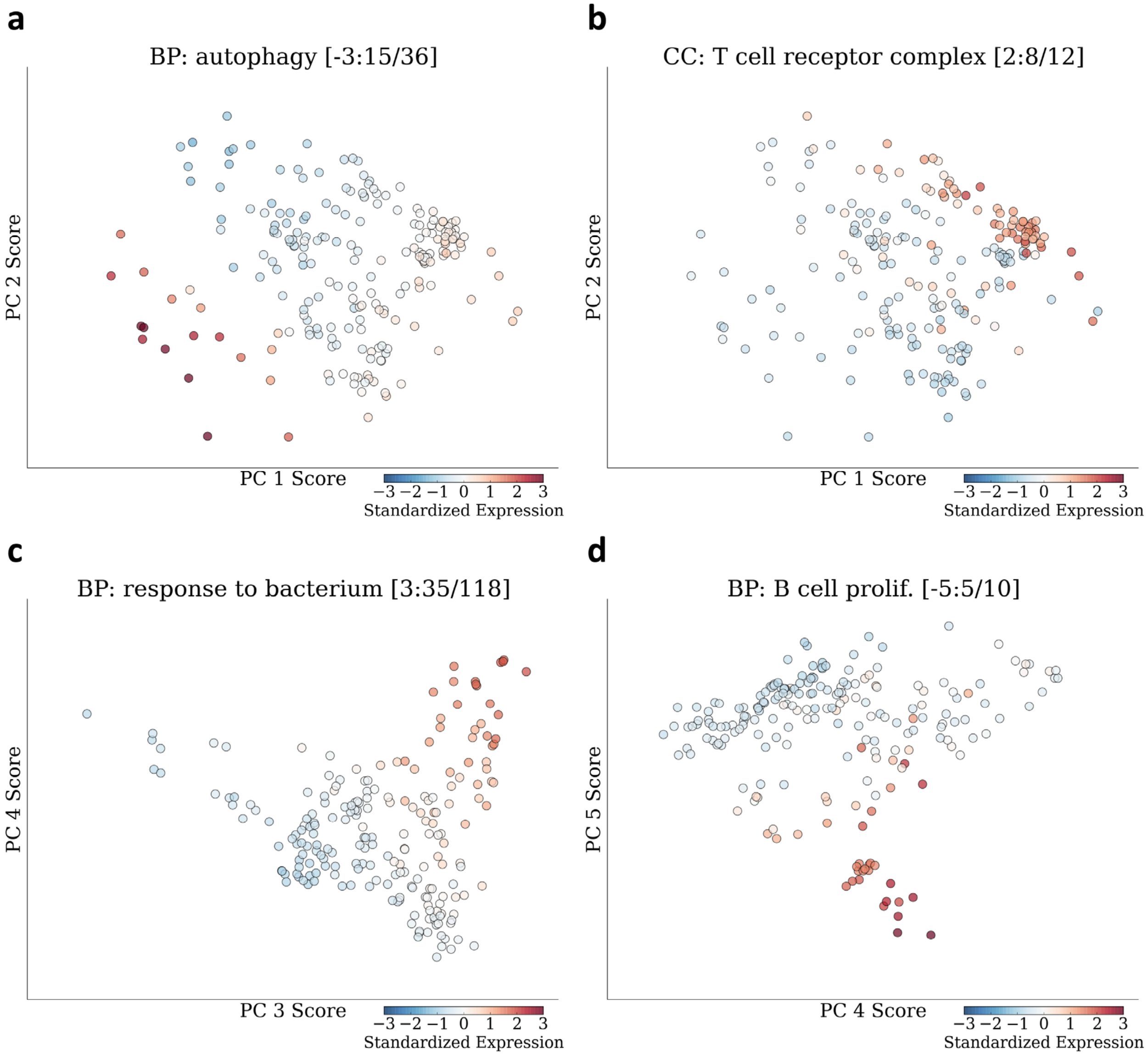

Figure 2: Signature Principal Component Plots. a-d Plots for various signatures and combinations of principal components, selected to highlight the relationship between the signatures and the individual components.
