# Supplementary material for: GO-PCA: An Unsupervised Method to Explore Gene Expression Data Using Prior Knowledge
Source: PLoS One. 2015 Nov 17;10(11):e0143196. doi: 10.1371/journal.pone.0143196 (PMC4648502; doi:10.1371/journal.pone.0143196)
Supplement: S3 Text — (PDF) [file pone.0143196.s015.pdf]

Supporting Text S3: The potential importance of ubiquitin ligases in reticulocyte development

Cullin-RING complexes constitute the largest class of ubiquitin ligases [1], and ubiquitylation is known to play an important role in mitophagy of damaged mitochondria, which involves recruitment of the E3 ubiquitin ligase PARK2 (parkin) [2]. While the role of ubiquitylation appears less well-studied in reticulocyte development, it is known to involve the ubiquitin-like protein LC3 [2], encoded by the gene *MAP1LC3A*. The DMAP dataset did not contain measurements for *PARK2*, nor for *MAP1LC3A*. However, LC3 is known to directly interact with SQSTM [3], one of the genes in the autophagy signature. The 18 genes in the ULC signature discovered by GO-PCA could therefore provide an opportunity for further research into the role of ubiquitin ligases in mitophagy.

References

[1] M. D. Petroski and R. J. Deshaies, "Function and regulation of cullin-RING ubiquitin ligases," *Nat. Rev. Mol. Cell Biol.*, vol. 6, no. 1, pp. 9–20, Jan. 2005.

[2] R. J. Youle and D. P. Narendra, "Mechanisms of mitophagy," *Nat. Rev. Mol. Cell Biol.*, vol. 12, no. 1, pp. 9–14, Jan. 2011.

[3] S. Pankiv, T. H. Clausen, T. Lamark, A. Brech, J.-A. Bruun, H. Outzen, A. Øvervatn, G. Bjørkøy, and T. Johansen, "p62/SQSTM1 binds directly to Atg8/LC3 to facilitate degradation of ubiquitinated protein aggregates by autophagy," *J. Biol. Chem.*, vol. 282, no. 33, pp. 24131–24145, Aug. 2007.
